# Kynurenine-AhR reduces T-cell infiltration and induces a delayed T-cell immune response by suppressing the STAT1-CXCL9/CXCL10 axis in tuberculosis

**DOI:** 10.1038/s41423-024-01230-1

**Published:** 2024-10-22

**Authors:** Xin Liu, Mengjie Yang, Ping Xu, Mingwei Du, Shanshan Li, Jin Shi, Qiang Li, Jinfeng Yuan, Yu Pang

**Affiliations:** 1 Department of Bacteriology and Immunology, Beijing Chest Hospital, Capital Medical University/Beijing Tuberculosis and Thoracic Tumor Research Institute, Beijing, China; 2 The Affiliated Infectious Diseases Hospital, Suzhou Medical College, Soochow University, Suzhou, China; 3 Department of Tuberculosis, Beijing Chest Hospital, Capital Medical University/Beijing Tuberculosis and Thoracic Tumor Research Institute, Beijing, China

**Keywords:** *Mycobacterium tuberculosis*, Tryptophan metabolism, IDO1, Immunosuppression, Chemokines, Immunology, Tuberculosis

## Abstract

Tuberculosis, caused by *Mycobacterium tuberculosis* (Mtb), is a critical global health issue that is complicated by the ability of the pathogen to delay the host’s T-cell immune response. This delay in T-cell recruitment to the site of infection is a pivotal survival strategy for Mtb, allowing it to establish a persistent chronic infection. To investigate the underlying mechanisms, this study focused on Mtb’s exploitation of host tryptophan metabolism. Mtb upregulates indoleamine 2,3-dioxygenase 1 (IDO1) in inflammatory macrophages, thereby increasing kynurenine (Kyn) production. Kyn then activates the aryl hydrocarbon receptor (AhR), leading to the upregulation of suppressor of cytokine signaling 3 and subsequent inhibition of the JAK-STAT1 signaling pathway. This results in reduced secretion of the chemokines CXCL9 and CXCL10, which are crucial for T-cell recruitment to the lungs. Supported by in vivo mouse models, our findings reveal that disrupting this pathway through AhR knockout significantly enhances T-cell infiltration and activity, thereby undermining Mtb-induced immunosuppression. In contrast, additional Kyn injection obviously inhibited T-cell infiltration and activity. These results highlight potential therapeutic targets of AhR and IDO1, offering new avenues for enhancing the host immune response against tuberculosis and guiding future vaccine development efforts.

## Introduction

*Mycobacterium tuberculosis* (Mtb) is the pathogen responsible for tuberculosis and can manipulate host responses to delay the recruitment of T cells to the lungs, thereby delaying the onset of adaptive immunity [1]. This delay is widely considered a major strategy employed by Mtb to establish its primary ecological niche and may represent a critical bottleneck for the eradication of Mtb by adaptive immunity [2].

During Mtb infection, adaptive immunity is crucial for controlling bacterial replication. CD4^+^ T cells play a pivotal role by secreting interferon-gamma (IFN-γ), which activates macrophages and enhances their bactericidal ability. The importance of CD4^+^ T cells in controlling tuberculosis infection has been confirmed in human immunodeficiency virus and tuberculosis coinfection models [3]. Additionally, CD8^+^ effector T cells (CTLs) can directly kill infected cells, thereby reducing the bacterial burden. In a macaque tuberculosis model, depletion of CD8^+^ T cells significantly impaired early control of Mtb infection [4]. To obtain successful immunotherapeutic strategies for Mtb control, we need to consider not only the activation status of T cells but also their infiltration rate at the site of infection. The limited success of tumor immunotherapy and adoptive T-cell transfer in restricting solid tumor growth highlights the importance of T-cell recruitment and infiltration in host immune responses [5, 6].

Many factors influence the arrival of T cells at the site of infection, including constraints on effective antigen presentation processes and the highly suppressive expansion of regulatory T-cell (Treg) populations, which limits the initiation and proliferation of effector T cells [7–9]. In the process of tuberculosis infection, chemokines secreted by inflammatory macrophages are also important factors affecting the infiltration of T cells into the site of infection [10].

The importance of tryptophan metabolism in inducing immune tolerance was initially established in the placenta [11]. However, increasing evidence suggests that tumor and pathogen pathways can exploit the mechanism responsible for immune tolerance to evade immune surveillance, thereby promoting the pathophysiology and progression of diseases [12–14]. Previous metabolomic analyses revealed that Mtb infection induces increased tryptophan metabolism in macrophages [15].

The tryptophan metabolite kynurenine (Kyn) acts as an effective negative regulator of inflammation and T-cell activity. Kyn possesses immunosuppressive properties, which are manifested through the promotion of T-cell apoptosis and the induction of Tregs [16, 17]. Additionally, studies have shown that Kyn can drive T-cell dysfunction through the Treg-macrophage suppressive axis by activating the aryl hydrocarbon receptor (AhR), a key signaling molecule [18]. The role of Kyn in inducing PD-1 has been confirmed in CTLs in an AhR-dependent manner [18, 19]. However, it is currently unclear whether Kyn can influence the recruitment of T cells by inflammatory macrophages, thereby affecting the adaptive immune response in tuberculosis. In this study, we found that Mtb promotes the production of Kyn by increasing the expression of indoleamine 2,3-dioxygenase 1 (IDO1) in inflammatory macrophages, thereby increasing the expression of intracellular AhR. AhR subsequently upregulates the expression of suppressor of cytokine signaling 3 (SOCS3), which inhibits the activation of the JAK-STAT1 pathway. This leads to a reduction in chemokine expression, thereby impairing the migration of T cells to the site of lung infection.

## Results

### High levels of Kyn inhibit T-cell infiltration in the lungs of patients with tuberculosis

**Fig. 1:**
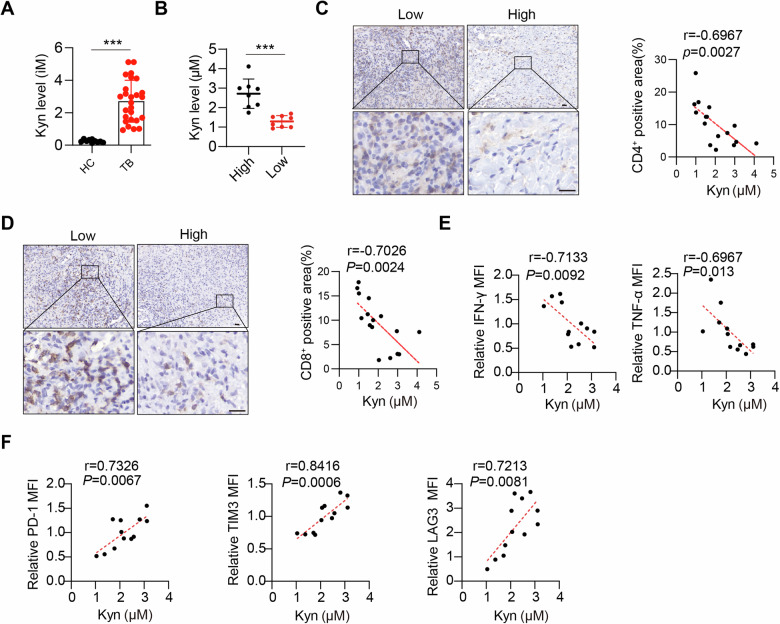
Kyn inhibits T-cell infiltration in tuberculosis patients. **A** LC‒MS detection of Kyn levels in the serum of healthy individuals (*n* = 10) and TB patients (*n* = 26). **B** Sixteen TB patients from group **A**, with corresponding lung tissue sections, were divided into high and low groups on the basis of the median Kyn levels. Lung sections from TB patients with high and low Kyn levels were immunohistochemically stained for CD4 (**C**) and CD8 (**D**). Scale bars, 20 μm (main image) and 20 μm (magnified region). Correlation between Kyn levels and CD4^+^ T-cell- or CD8^+^ T-cell-positive areas in human TB lung tissues. Each data point represents the value from an individual patient (*n* = 16). **E** The correlation between Kyn levels and the expression of the T-cell effector cytokines IFN-γ and TNF-α in people with TB. Each data point represents the value from an individual patient (*n* = 12). **F** The correlation between Kyn levels and the expression levels of the T-cell inhibitory receptors PD-1, TIM3, and LAG3 in T cells from TB patients. Each data point represents a value from an individual patient (*n* = 12). Statistical significance was measured by Spearman’s correlation test in **C**–**F**. The data are presented as the means ± SDs. *p* values were calculated via unpaired two-tailed Student’s *t* test. ***p < 0.001

Mtb infection may lead to metabolic reprogramming of host cells, including alterations in tryptophan metabolism [15]. This metabolic reprogramming could affect the host cell’s defense mechanisms against Mtb. Compared with healthy controls, patients with active tuberculosis have decreased serum levels of tryptophan and increased levels of its metabolite (Kyn). Consistent with previous research findings [20], our comparison of Kyn expression levels between healthy individuals and patients with active tuberculosis revealed a significant elevation in serum Kyn levels in patients (Fig. 1A). T cells are essential for immunity against Mtb, and recruiting them to the lungs is necessary to confine Mtb within granulomas [21]. To assess the impact of Kyn on CD4^+^ and CD8^+^ T-cell function, we divided tuberculosis patients into high- and low-Kyn-expressing groups (Fig. 1B). Our findings revealed a significant decrease in the number of lung tissue-infiltrating CD4^+^ and CD8^+^ T cells in patients with high Kyn expression in the serum compared with those in patients with low Kyn expression (Fig. 1C, D). These findings suggest that Kyn may influence the recruitment of effector T cells to the lungs. Furthermore, we observed a negative correlation between Kyn levels in patient serum and the expression of the effector cytokines IFN-γ and TNF-α by T cells (Fig. 1E). Additionally, there was a positive correlation between Kyn levels and the expression levels of inhibitory receptors (IRs), including PD-1, TIM3, and LAG3, in peripheral blood T cells (Fig. 1F). These results suggest that during tuberculosis infection, Mtb induces host tryptophan metabolism to produce a large amount of the metabolite Kyn, thereby affecting the infiltration level and function of effector T cells in the lungs, thus achieving immune evasion.

### Mtb inhibits T-cell infiltration through an increase in the inflammatory macrophage IDO1-Kyn metabolic pathway

**Fig. 2:**
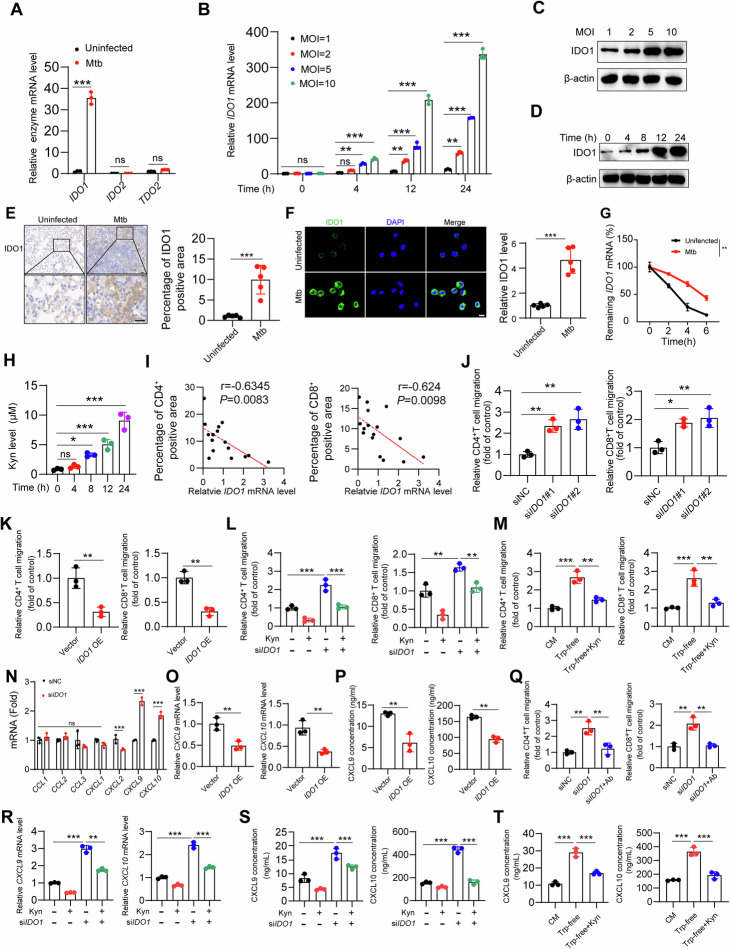
Mtb inhibits T-cell infiltration by activating the IDO1-Kyn pathway. **A** qPCR analysis of the indicated mRNAs in THP-1 cells pretreated with IFN-γ (20 ng/mL) for 24 h and infected with or without Mtb (MOI = 5) for 24 h. **B** THP-1 cells were pretreated with IFN-γ (20 ng/mL) for 24 h and then infected with different MOIs of Mtb for the indicated hours. *IDO1* mRNA expression was measured via qPCR analysis. **C** THP-1 cells were pretreated with IFN-γ (20 ng/mL) for 24 h and then infected with different MOIs of Mtb. The indicated antibodies were used for western blot analysis. **D** THP-1 cells were pretreated with IFN-γ (20 ng/mL) for 24 h and then infected with Mtb for the indicated hours. The indicated antibodies were used for western blot analysis. **E** Lung sections from mice infected with or without Mtb for 3 weeks were immunohistochemically stained with IDO1. Scale bars, 20 μm (main image) and 20 μm (magnified region). **F** Alveolar macrophages from infected or uninfected mice were stained for IDO1 and analyzed via confocal microscopy. Scale bars, 20 μm. **G** THP-1 cells pretreated with IFN-γ (20 ng/mL) for 24 h were infected with or without Mtb (MOI = 5) for 12 h. The cells were treated with actinomycin D (10 μg/ml). The *IDO1* mRNA levels that remained after treatment (as a percentage of the starting *IDO1* mRNA levels.) were examined at the indicated time points. One-way repeated-measures ANOVA test. **H** LC‒MS detection of Kyn levels in THP-1 cells infected with Mtb for the indicated hours. **I** Correlations between *IDO1* mRNA expression and CD4^+^ T-cell and CD8^+^ T-cell infiltration in the lungs of tuberculosis patients. **J** THP-1 cells were transfected with NC or *IDO1* siRNA for 24 h, followed by IFN-γ (20 ng/mL) treatment for 24 h. CD4^+^ and CD8^+^ T-cell infiltration was measured via a transwell migration assay. **K** THP-1 cells were transfected with or without IDO1 via jetPRIME, followed by IFN-γ (20 ng/mL) treatment for 24 h. Then, CD4^+^ and CD8^+^ T-cell infiltration was measured via a transwell migration assay. **L** The same as in **J**, except that the cells were treated with or without Kyn (100 μM) for 24 h. CD4^+^ and CD8^+^ T-cell migration was measured via **a** transwell migration assay. **M** THP-1 cells were cultured in complete medium and tryptophan-free medium with or without Kyn (100 μM) for 24 h. CD4^+^ and CD8^+^ T-cell migration was measured via a transwell migration assay. **N** As in **J**, qPCR analysis of the indicated mRNAs was subsequently performed. **O**, **P** As in **K**, the mRNA (**O**) and protein expression (**P**) of the indicated genes were measured. **Q** The same as **J**, except that the cells were treated with or without anti-CXCL9 and anti-CXCL10 neutralizing antibodies for 24 h. CD4^+^ and CD8^+^ T-cell infiltration was measured via a transwell migration assay. **R**, **S** Similar to **L**, the mRNA (**R**) and protein (**S**) expression levels of the indicated genes were subsequently measured. **T** As in **M**, ELISA of CXCL9/10 was subsequently performed. The data are presented as the means ± SDs. *p* values were calculated via one-way ANOVA (**B**, **H**, **J**, **L**, **M**, **Q**–**T**), unpaired two-tailed Student’s *t* test (**A**, **E**, **F**, **K**, **N**, **O**, **P**) or Spearman’s correlation test in **I**. *p < 0.05, **p < 0.01, ***p < 0.001; ns not significant (*p* > 0.05)

Inflammatory macrophages play a crucial role in recruiting T cells to the site of infection [22]. We investigated the expression levels of several tryptophan-degrading enzymes that convert tryptophan metabolism into the metabolite Kyn in inflammatory macrophages. Interestingly, we found that after infection with Mtb, the expression level of only *IDO1* significantly increased in inflammatory macrophages, whereas other tryptophan-degrading enzymes, such as *IDO2* and *TDO2*, did not significantly change (Fig. 2A, Supplementary Fig. 1A). We first investigated whether the expression level of IDO1 in macrophages is induced in an Mtb burden-dependent manner. The results revealed that IDO1 levels increased with both the Mtb multiplicity of infection (MOI) and the duration of infection (Fig. 2B–D, Supplementary Fig. 1B, C). Similarly, compared with those of uninfected mice, the lung tissues of Mtb-infected mice also presented increased expression of IDO1 (Fig. 2E), particularly in alveolar macrophages (Fig. 2F). Previous studies have reported that colon cancer cells can increase IDO1 expression by increasing the stability of *IDO1* mRNA [23]. We hypothesize that Mtb may promote IDO1 expression by increasing the stability of *IDO1* mRNA. In the presence of actinomycin D, the degradation rate of *IDO1* mRNA in Mtb-infected cells was significantly lower than that in uninfected cells (Fig. 2G). This result demonstrates that Mtb promotes IDO1 expression by increasing *IDO1* mRNA stability. Using a liquid chromatography mass spectrometer (LC‒MS), we examined the levels of Kyn inside macrophages before and after Mtb infection. We found that Mtb infection led to significant upregulation of IDO1 accompanied by a substantial increase in intracellular Kyn production (Fig. 2H, Supplementary Fig. 1D). Correlation analysis revealed that the expression levels of *IDO1* mRNA in the lungs of tuberculosis patients were negatively correlated with the infiltration of CD4^+^ T and CD8^+^ T cells (Fig. 2I). In vitro T-cell migration assays confirmed that knocking down *IDO1* in inflammatory macrophages increased the migration of both CD4^+^ and CD8^+^ T cells compared with that in the control group (Fig. 2J, Supplementary Fig. 1E). Conversely, overexpression of *IDO1* inhibited the migration of both CD4^+^ and CD8^+^ T cells (Fig. 2K, Supplementary Fig. 1F). To further investigate whether IDO1 affects T-cell migration by producing Kyn from tryptophan metabolism, we supplemented the culture medium with additional Kyn. Interestingly, we found that Kyn reversed the *IDO1* knockdown-induced migration of CD4^+^ and CD8^+^ T cells (Fig. 2L, Supplementary Fig. 1G). Similarly, under tryptophan-deficient conditions, inflammatory macrophages promoted the migration of CD4^+^ and CD8^+^ T cells in vitro; however, this phenomenon could be abrogated by additional supplementation with Kyn (Fig. 2M, Supplementary Fig. 1H). Chemokines play crucial roles in guiding T cells to sites of infection in the lungs [24]. Therefore, we investigated whether Mtb influences the expression of chemokines by upregulating IDO1 expression in macrophages, thereby reducing T-cell infiltration at the site of infection. We examined a series of key chemokines expressed by macrophages that are involved in T-cell recruitment. The results revealed that knocking down *IDO1* significantly increased the expression levels of *CXCL9* and *CXCL10* in inflammatory macrophages, whereas other chemokines, such as *CCL1* and *CCL2*, did not significantly change (Fig. 2N). Additionally, correlation analysis of *CXCL9/10* mRNA levels and CD4^+^ and CD8^+^ T-cell infiltration in the lungs of tuberculosis patients revealed the significant role of CXCL9/10 expression in T-cell infiltration (Supplementary Fig. 1I, J). Upon overexpression of *IDO1* in inflammatory macrophages, the expression levels of CXCL9 and CXCL10 decreased (Fig. 2O‒P, Supplementary Fig. 1K). Treatment with neutralizing antibodies against CXCL9 and CXCL10 in inflammatory macrophages negated the enhanced T-cell migration observed upon *IDO1* knockdown (Fig. 2Q, Supplementary Fig. 1L). However, in *IDO1*-deficient inflammatory macrophages, the addition of Kyn resulted in a reduction in the mRNA levels of *CXCL9/10* (Fig. 2R, Supplementary Fig. 1M). ELISAs further confirmed that knocking down *IDO1* followed by Kyn supplementation decreased the protein levels of CXCL9 and CXCL10 (Fig. 2S). Furthermore, as expected, inflammatory macrophages cultured under tryptophan-deficient conditions presented elevated levels of CXCL9/10 expression; however, this phenomenon was reversed by additional supplementation with Kyn (Fig. 2T, Supplementary Fig. 1N). CXCR3 is the receptor for CXCL9 and CXCL10 and is expressed primarily on CD4^+^ and CD8^+^ T cells. CXCR3 is rapidly induced after the activation of naive T cells and is highly expressed on effector T cells. We detected the expression of CXCR3 in the lungs of Mtb-infected mice treated with or without Kyn through flow cytometry. The results showed that Kyn does not affect the expression of CXCR3 on CD4^+^ T cells or CD8^+^ T cells (Supplementary Fig. 1O‒P). These findings suggest that IDO1 inhibits the production of CXCL9 and CXCL10 by metabolizing tryptophan to Kyn, thereby suppressing T-cell migration.

### IDO1 inhibits T-cell infiltration through the STAT1-CXCL9/10 signaling pathway

**Fig. 3:**
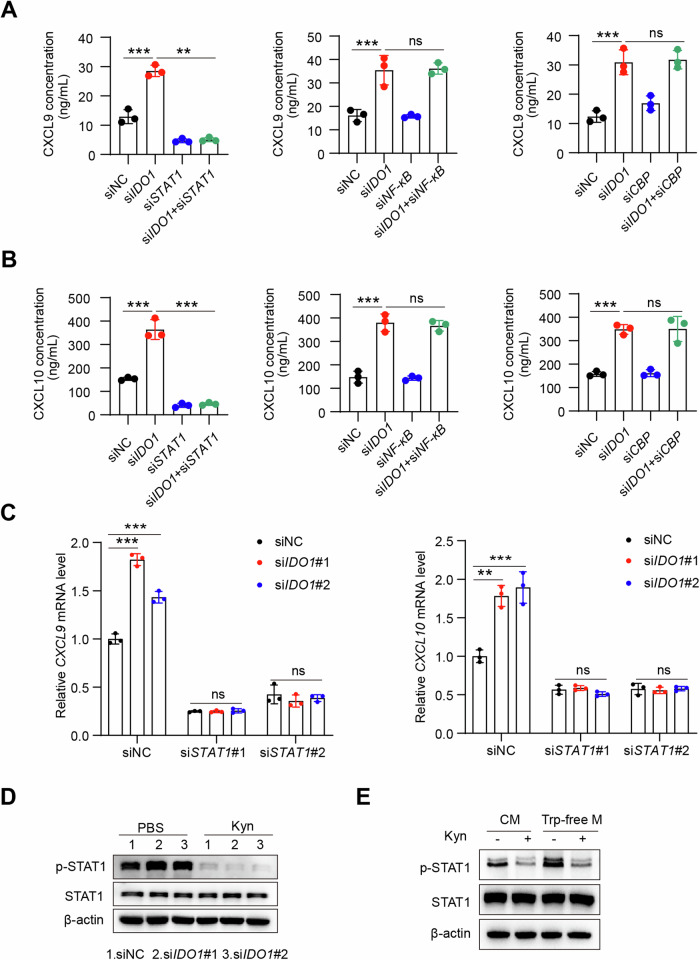
Mtb suppresses CXCL9/10 expression by inhibiting the STAT1 pathway. **A**, **B** THP-1 cells were transfected with NC, *IDO1*, *STAT1*, *NF-κB*, or *CBP* siRNA for 24 h, followed by IFN-γ (20 ng/mL) treatment for 24 h. Then, an ELISA of CXCL9/10 was performed. **C** THP-1 cells were transfected with NC, *IDO1*, or cotransfected with *IDO1* and *STAT1* siRNA for 24 h, followed by IFN-γ (20 ng/mL) treatment for 24 h. Then, qPCR of the indicated genes was performed. **D** THP-1 cells were transfected with NC or *IDO1* siRNA for 24 h, treated with IFN-γ (20 ng/mL) for 24 h, and then treated with or without Kyn (100 μM) for 24 h. Then, the indicated antibodies were measured via western blot analysis. **E** THP-1 cells were cultured in complete medium and tryptophan-free medium with or without Kyn (100 μM) for 24 h, after which the indicated antibodies were used for western blot analysis. The data are presented as the means ± SDs. *p* values were calculated via one-way ANOVA. **p < 0.01, ***p < 0.001; ns not significant (*p* > 0.05)

STAT1, NF-κB, and CREB (adenosine 3′,5′-monophosphate response element–binding protein)–binding protein (CBP) have been reported as transcription factors that mediate CXCL9 and CXCL10 transcription [25–28]. These results indicate that knocking down *STAT1*, rather than *NF-κB* or *CBP*, can abolish the elevated protein levels of CXCL9 and CXCL10 induced by *IDO1* knockdown (Fig. 3A, B). qPCR experiments also confirmed that knocking down *STAT1* eliminated the increased mRNA levels of *CXCL9* and *CXCL10* caused by *IDO1* knockdown (Fig. 3C, Supplementary Fig. 2A, B). After knocking down *IDO1* in inflammatory macrophages, the baseline abundance of STAT1 remained unchanged, but there was an increase in STAT1 tyrosine phosphorylation. However, this effect was reversed upon the addition of Kyn (Fig. 3D, Supplementary Fig. 2C). Similarly, under tryptophan-deficient conditions, results similar to those of *IDO1* knockdown were observed (Fig. 3E, Supplementary Fig. 2D). These findings suggest that Mtb enhances the expression of the tryptophan-metabolizing enzyme IDO1 and utilizes its metabolite Kyn to inhibit the STAT1-CXCL9/10 signaling pathway.

### The IDO1-Kyn metabolic pathway diminishes the phosphorylation of STAT1 through AhR activation

AhR is a ligand-dependent transcription factor crucial for a wide range of immune functions, including regulating immune cell differentiation and inflammatory responses and maintaining populations of innate and adaptive response cells [29–31]. Previous studies have suggested that Kyn is an endogenous ligand for AhR [32]. Upon ligand binding, AhR translocates into the cell nucleus and upregulates the expression of two AhR target genes, genes encoding cytochrome P450 family 1 subfamily A polypeptide 1 (CYP1A1) and cytochrome P450 family 1 subfamily B polypeptide 1 (CYP1B1), to exert its biological effects [33].

**Fig. 4:**
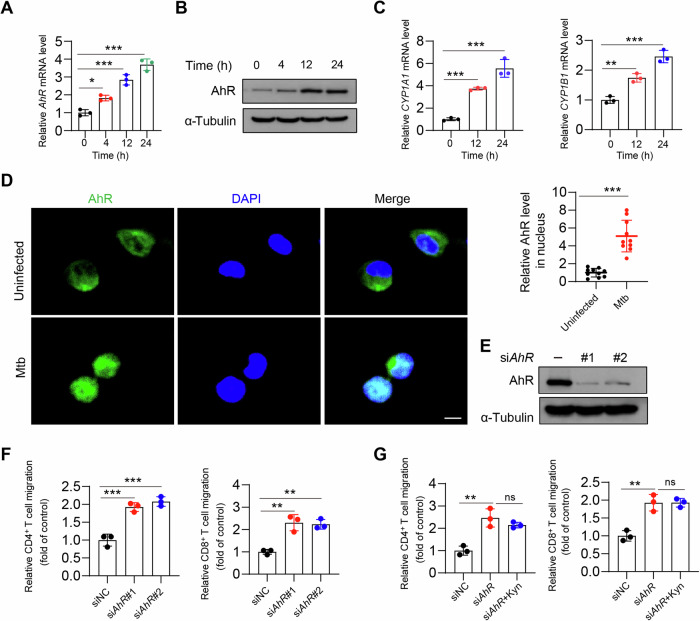
Mtb activates AhR to inhibit T-cell migration. **A**–**C** THP-1 cells were pretreated with IFN-γ (20 ng/mL) for 24 h and then infected with Mtb (MOI = 5) for the indicated hours. The mRNA (**A** and **C**) and protein expression (**B**) of the indicated genes were measured. **D** As in **A**, the cells were subsequently stained for AhR and analyzed via confocal microscopy. Scale bars, 20 μm. **E** THP-1 cells were transfected with NC or *AhR* siRNA for 48 h, after which the indicated antibodies were used for western blot analysis. **F** THP-1 cells were transfected with NC or *AhR* siRNA for 24 h, followed by IFN-γ (20 ng/mL) treatment for 24 h. Then, CD4^+^ and CD8^+^ T-cell infiltration was measured via a transwell migration assay. **G** The same as in **F**, except that the cells were treated with or without Kyn (100 μM) for 24 h. Then, CD4^+^ and CD8^+^ T-cell migration was measured via **a** transwell migration assay. The data are presented as the means ± SDs. *p* values were calculated via one-way ANOVA (**A**, **C**, **F**, **G**) or unpaired two-tailed Student’s *t* test (**D**). *p < 0.05, **p < 0.01, ***p < 0.001; ns not significant (*p* > 0.05)

**Fig. 5:**
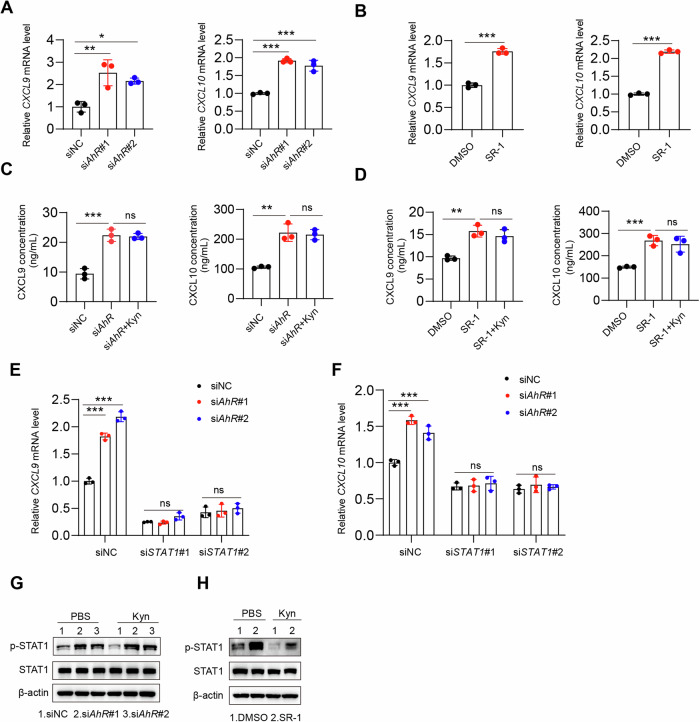
Mtb activates AhR to attenuate the STAT1-CXCL9/10 pathway. **A** THP-1 cells were transfected with NC or *AhR* siRNA for 24 h, followed by IFN-γ (20 ng/mL) treatment for 24 h. qPCR of the indicated genes was subsequently performed. **B** THP-1 cells were pretreated with IFN-γ (20 ng/mL) for 24 h, followed by treatment with DMSO or SR-1 (1 μM) for 24 h. qPCR of the indicated genes was subsequently performed. **C** The same as in **A**, except that the cells were treated with or without Kyn (100 μM) for 24 h. ELISA of CXCL9/10 was subsequently performed. **D** The same as in **B**, except that the cells were treated with or without Kyn (100 μM) for 24 h. ELISA of CXCL9/10 was subsequently performed. **E**, **F** THP-1 cells were transfected with NC or *AhR* or cotransfected with *AhR* and *STAT1* siRNA for 24 h, followed by IFN-γ (20 ng/mL) treatment for 24 h. Then, qPCR of the indicated genes was performed. **G** The same as in **A**, except that the cells were treated with or without Kyn (100 μM) for 24 h. Then, the indicated antibodies were used for western blot analysis. **H** The same as in **B**, except that the cells were treated with or without Kyn (100 μM) for 24 h. Then, the indicated antibodies were used for western blot analysis. The indicated antibodies were subsequently used for western blot analysis. The data are presented as the means ± SDs. *p* values were calculated via one-way ANOVA **(A**, **C**–**F**) or an unpaired two-tailed Student’s *t* test (**B**). *p < 0.05, **p < 0.01, ***p < 0.001; ns not significant (*p* > 0.05)

To investigate the role of the transcription factor AhR in the Mtb-mediated regulation of immune evasion processes, we found that the expression of *AhR*, *CYP1A1*, and *CYP1B1* was significantly upregulated in Mtb-infected inflammatory macrophages compared with that in uninfected inflammatory cells (Fig. 4A–C, Supplementary Fig. 3A, B). Consistent with these results, the results of the immunofluorescence experiments indicated that AhR translocated from the cytoplasm to the cell nucleus after Mtb infection (Fig. 4D, Supplementary Fig. 3C). Next, we attempted to elucidate whether AhR is involved in the tryptophan‒Kyn metabolic pathway to regulate the chemotaxis of inflammatory macrophages toward T cells. We found that the knockdown of *AhR* promoted the chemotactic effect of inflammatory macrophages on T cells (Fig. 4E, F, Supplementary Fig. 3D). However, the addition of exogenous Kyn was unable to abolish this chemotactic effect (Fig. 4G, Supplementary Fig. 3E). To further explore the impact of AhR on the STAT1‒CXCL9‒10 pathway, we observed that inflammatory macrophages presented significantly increased levels of CXCL9 and CXCL10 following AhR inhibition (Fig. 5A, B, Supplementary Fig. 3F). However, the addition of exogenous Kyn did not affect the levels of CXCL9/10 (Fig. 5C, D, Supplementary Fig. 3G, H). In inflammatory macrophages, *STAT1* knockdown ameliorated the *AhR*-induced increase in the mRNA levels of *CXCL9* and *CXCL10* (Fig. 5E, F). Knockdown of *AhR* or the use of the AhR inhibitor SR-1 also promoted the phosphorylation of STAT1; however, the addition of exogenous Kyn was unable to suppress phosphorylated STAT1 (Fig. 5G, H). Similar results were also obtained from BMDMs (Supplementary Fig. 3I, J). These results suggest that the IDO1-Kyn metabolic pathway activates AhR, leading to the suppression of STAT1 activation and the expression of its downstream effector molecules.

### AhR diminishes the phosphorylation of JAK-STAT1 by upregulating SOCS3

**Fig. 6:**
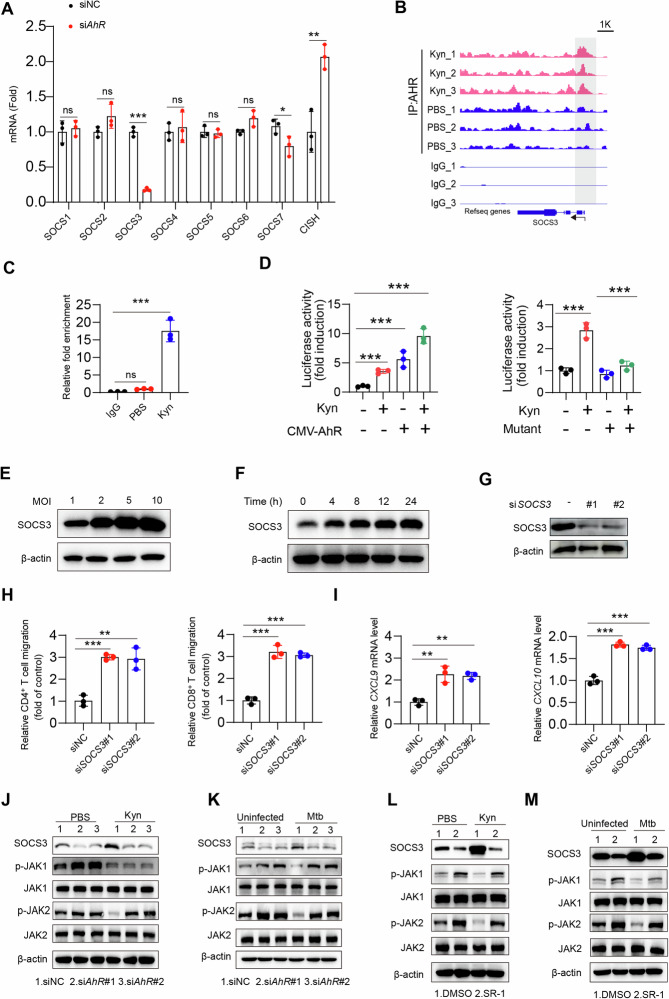
AhR regulates the JAK-STAT1 pathway by upregulating SOCS3. **A** THP-1 cells were transfected with NC or *AhR* siRNA for 24 h, followed by IFN-γ (20 ng/mL) treatment for 24 h. qPCR of the indicated genes was subsequently performed. **B** CUT&Tag analysis was performed with an antibody against AhR in THP-1 cells pretreated with IFN-γ (20 ng/mL) for 24 h and then with PBS or Kyn for 24 h. Representative CUT&Tag-seq tracks at the *SOCS3* gene locus. **C** ChIP‒qPCR analysis was performed with an antibody against the AhR and SOCS3 promoter-specific primers. **D** THP-1 cells were cotransfected with the wild-type or mutated *SOCS3* promoter-luciferase reporter PGL4.10 and *AhR*-overexpressing pCMV-6 for 24 h. The cells were treated with Kyn (100 μM) for another 24 h, followed by luciferase activity analysis. **E** THP-1 cells were pretreated with IFN-γ (20 ng/mL) for 24 h and then infected with different MOIs of Mtb. The indicated antibodies were used for western blot analysis. **F** THP-1 cells were pretreated with IFN-γ (20 ng/mL) for 24 h and then infected with Mtb for the indicated hours. The indicated antibodies were used for western blot analysis. **G** THP-1 cells were transfected with NC or *SOCS3* siRNA for 48 h, and the indicated antibodies were subsequently used for western blot analysis. **H** THP-1 cells were transfected with NC or *SOCS3* siRNA for 24 h, followed by IFN-γ (20 ng/mL) treatment for 24 h. Then, CD4^+^ and CD8^+^ T-cell migration was measured via a transwell migration assay. **I** As in **G**, qPCR of the indicated genes was subsequently performed. **J** THP-1 cells were transfected with NC or *AhR* siRNA for 48 h, followed by IFN-γ (20 ng/mL) treatment for 24 h. Then, the cells were treated with or without Kyn (100 μM) for 24 h. Then, the indicated antibodies were used for western blot analysis. **K** THP-1 cells were transfected with NC or AhR siRNA for 48 h, followed by IFN-γ (20 ng/mL) treatment for 24 h. Then, the cells were infected with or without Mtb (MOI = 5) for 24 h. The indicated antibodies were subsequently used for western blot analysis. **L** THP-1 cells were pretreated with IFN-γ (20 ng/mL) for 24 h, followed by Kyn (100 μM) or Kyn combined with SR-1 (1 μM) for 24 h. Then, the indicated antibodies were used for western blot analysis. **M** THP-1 cells were pretreated with IFN-γ (20 ng/mL) for 24 h, followed by infection with Mtb (MOI = 5) combined with SR-1 (1 μM) for 24 h. Then, the indicated antibodies were used for western blot analysis. The data are presented as the means ± SDs. *p* values were calculated via one-way ANOVA (**C**, **D**, **H**, **I**) or an unpaired two-tailed Student’s *t* test (**A**). *p < 0.05, **p < 0.01, ***p < 0.001; ns not significant (*p* > 0.05)

Next, we explored the precise molecular mechanism by which AhR inhibits STAT1 signaling. The cytokine signaling suppressor protein (SOCS) acts as a direct negative regulator of the JAK-STAT pathway [34]. The SOCS family includes SOCS1-7 and CISH. Furthermore, upon *AhR* knockdown in macrophages, only the SOCS3 mRNA expression level was significantly downregulated (Fig. 6A). We hypothesized that AhR might trigger the expression of SOCS3 in macrophages in response to Mtb infection. We further discovered that the SOCS3 promoter contains an AhR-binding core sequence (5’-GCGTG), as indicated by the JASPAR database. The results of the ChIP‒qPCR and CUT&Tag analyses demonstrated that the binding of AhR to the SOCS3 promoter was greater in the Kyn-treated groups than in the PBS- or IgG-treated groups (Fig. 6B, C). Moreover, dual-luciferase reporter assays indicated that the binding of AhR to the SOCS3 promoter increased reporter gene expression (Fig. 6D). However, this effect was abolished when the 5’-GCGTG core sequence in the SOCS3 promoter was mutated to 5’-GCATG (Fig. 6D). With increasing time and an increase in the number of Mtb, the expression level of SOCS3 also increased (Fig. 6E, F, Supplementary Fig. 4A, B).

Furthermore, when *SOCS3* was knocked down via siRNA (Fig. 6G), inflammatory macrophages presented increased T-cell migration capacity (Fig. 6H, Supplementary Fig. 4C) accompanied by the upregulation of CXCL9/10 (Fig. 6I, Supplementary Fig. 4D). Furthermore, after Kyn or Mtb treatment, the expression of SOCS3 markedly increased compared with that in the control group, but Kyn treatment or Mtb infection did not alter SOCS3 expression or target protein phosphorylation of JAK1 or JAK2 in AhR-silenced macrophages (Fig. 6J, K, Supplementary Fig. 4E, F).

Consistently, the enhancing effect of Kyn or Mtb infection on SOCS3 was abolished when the cells were pretreated with the AhR inhibitor SR-1 (Fig. 6L, M). These findings collectively suggest that Kyn-AhR initiates SOCS3, thereby regulating the JAK-STAT1 signaling pathway to impact host immunity.

### Tryptophan-Kyn metabolism inhibition leads to early recruitment of T cells to the lung during Mtb infection

**Fig. 7:**
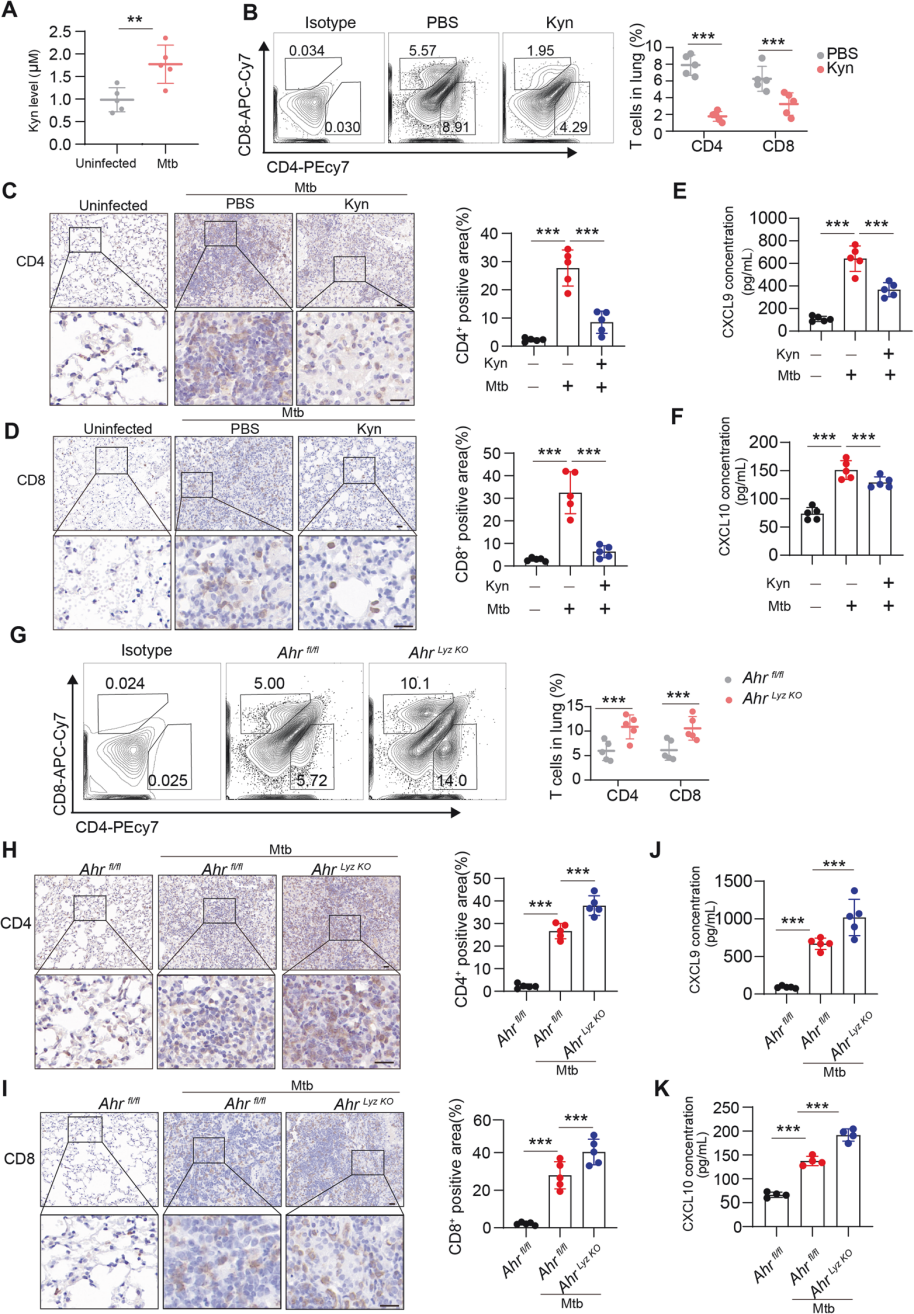
The Kyn–AhR pathway diminished host resistance to Mtb infection in vivo. **A** Serum Kyn levels of C57BL/6J mice that were not infected or infected with the indicated Mtb strains via aerosols (~100 CFUs) for 3 weeks were subsequently measured via LC‒MS (*n* = 5 mice per group). **B**–**F** C57BL/6J mice were left uninfected or infected with the indicated Mtb strains by aerosol (~100 CFUs), and then, the Mtb-infected C57BL/6J mice were treated with PBS or Kyn daily starting one day after infection for 3 weeks (*n* = 5 mice per group). The lungs were subsequently harvested at 3 weeks. **B** Single-cell suspensions of lungs from the two treatment groups were stained with appropriate antibodies and analyzed via multicolor flow cytometry. Flow cytometric analysis of the expression of CD4^+^ and CD8^+^ cells. Immunohistochemical analysis of CD4^+^ (**C**) and CD8^+^ (**D**) lung sections was performed. Scale bars, 20 μm (main image) and 20 μm (magnified region). **E**, **F** The serum concentration of CXCL9/10 was determined via an ELISA Kit. **G**–**K** *Ahr*^*fl/fl*^ mice and *Ahr*^*Lyz-KO*^ mice were left uninfected or infected with the indicated Mtb strains by aerosol (~100 CFUs), after which the lungs were harvested at 3 weeks (*n* = 5 mice per group). **G** Single-cell suspensions of lungs from the two treatment groups were stained with appropriate antibodies and analyzed via multicolor flow cytometry. Flow cytometric analysis of the expression of CD4^+^ and CD8^+^ T cells. Immunohistochemical analysis of CD4^+^ (**H**) and CD8^+^ (**I**) lung sections was performed. Scale bars, 20 μm (main image) and 20 μm (magnified region). **J**, **K** Serum levels of CXCL9/10 were measured with an ELISA kit. The data are presented as the means ± SDs. *p* values were calculated via one-way ANOVA (**C**–**F**, **H**–**K**) or an unpaired two-tailed Student’s *t* test (**A**, **B**, **G**). **p < 0.01, ***p < 0.001

**Fig. 8:**
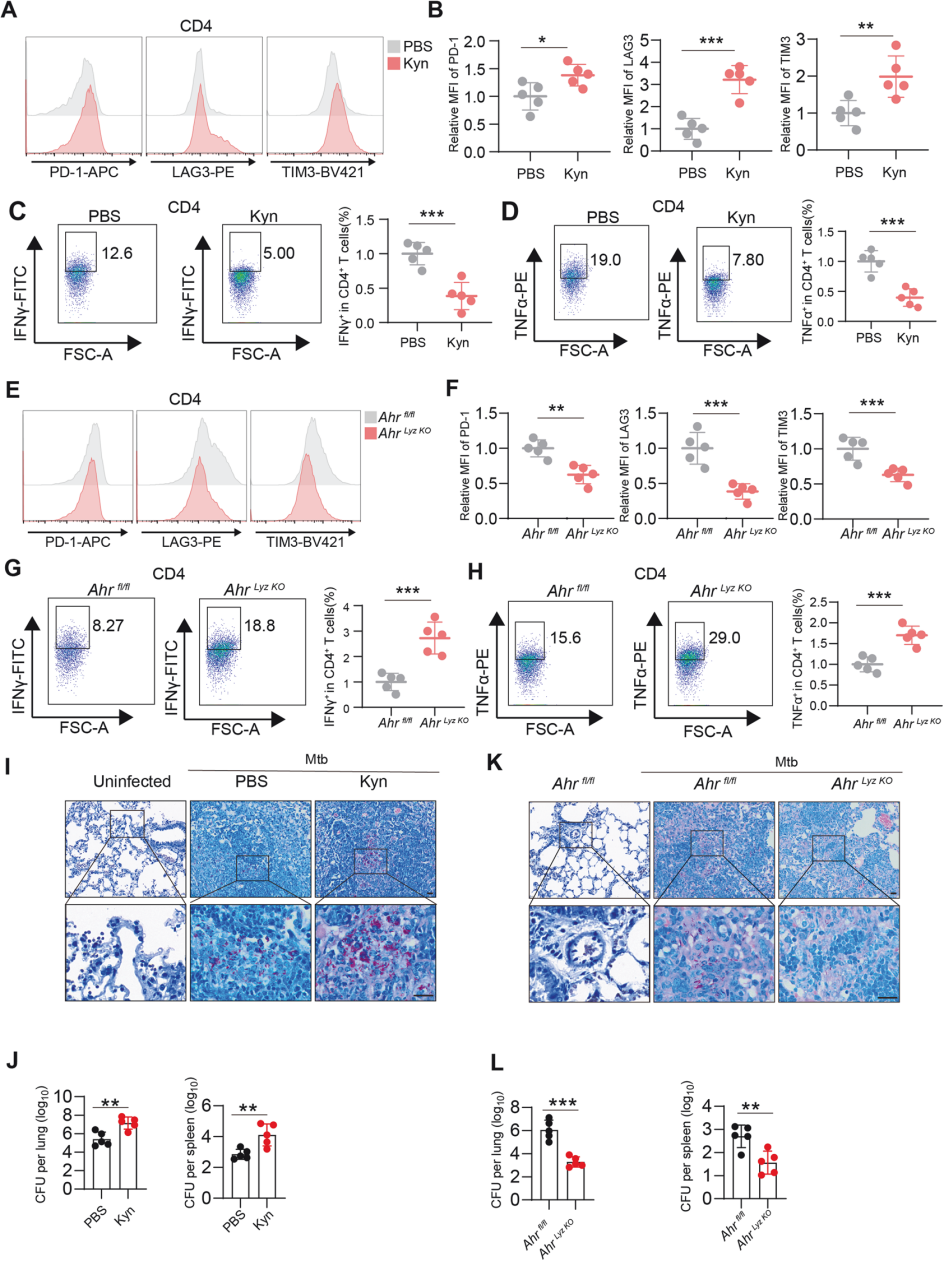
The Kyn–AhR pathway leads to CD4^+^ T-cell dysfunction. **A**–**D** Mtb-infected C57BL/6J mice were treated with PBS or Kyn (20 mg/kg) daily starting one day after infection (*n* = 5 mice per group). The mice were sacrificed after 6 weeks, and the lungs and spleens were harvested. Single-cell suspensions of lungs from the two treatment groups were stained with appropriate antibodies and analyzed via multicolor flow cytometry. **A**, **B** We detected differences in the frequencies or geometric mean fluorescence intensities (gMFIs) of PD-1, LAG3, and TIM3 expression on activated CD4^+^ T cells. **C** IFN-γ expression on activated CD4^+^ T cells. **D** TNF-α expression on activated CD4^+^ T cells. **E**–**H** Mtb-infected *Ahr*^*fl/fl*^ and *Ahr*^*Lyz-KO*^ mice were sacrificed after 6 weeks, and the lungs were harvested (*n* = 5 mice per group). Single-cell suspensions of lungs from the two treatment groups were stained with appropriate antibodies and analyzed via multicolor flow cytometry. **E**, **F** We detected differences in the frequency or gMFI of PD-1, LAG3, and TIM3 expression on activated CD4^+^ T cells. **G** IFN-γ expression in activated CD4^+^ T cells. **H** TNF-α expression in activated CD4^+^ T cells. gMFI is mostly used for low-abundance cell surface markers and transcription factors. **I** As in **A**–**D**, acid‒fast staining of lung sections was subsequently performed. Scale bars, 20 μm (main image) and 20 μm (magnified region). **J** Bacterial load in homogenates from the lungs or spleens of the mice treated as described in **A**–**D**. **K** As in **E**–**H**, acid‒fast staining of lung sections was subsequently performed. Scale bars, 20 μm (main image) and 20 μm (magnified region). **L** Bacterial load in homogenates from the lungs or spleens of the mice treated as described in **E**–**H**. The data are presented as the means ± SDs. *p* values were calculated via two-tailed Student’s *t* test. *p < 0.05, **p < 0.01, ***p < 0.001

The preceding experiments indicated that Mtb promotes Kyn-AhR signaling in macrophages to inhibit T-cell infiltration in vitro. Here, we further validated this process in vivo. We first investigated the impact of the Kyn-AhR signaling pathway on T cells within the lungs during Mtb infection in a mouse model. Although serum Kyn levels were elevated in Mtb-infected mice compared with uninfected mice (Fig. 7A), compared with PBS, treatment with Kyn in Mtb-infected mice led to a significant reduction in lung CD4^+^ and CD8^+^ T cells (Fig. 7B–D). Similarly, the expression levels of CXCL9 and CXCL10 in the serum were decreased (Fig. 7E, F). However, in *Ahr* ^*Lyz-KO*^ mice, the infiltration levels of CD4^+^ and CD8^+^ T cells in the lungs were significantly greater than those in WT mice (Fig. 7G–I), and the expression levels of CXCL9 and CXCL10 in the serum were elevated (Fig. 7J, K). The delayed immune response of effector T cells is an immunological characteristic of the lung. Additionally, during chronic infection, Mtb induces host T-cell dysfunction, which is another important immunological feature [35]. Injecting Kyn into Mtb-infected mice resulted in the upregulation of IRs (Fig. 8A, B, Supplementary Fig. 5A–C) but reduced the release of the effector molecules IFN-γ and TNF-α from CD4^+^ and CD8^+^ T cells (Fig. 8C, D, Supplementary Fig. 5D, E). Furthermore, compared with those in *Ahr*^*fl/fl*^ mice, the expression levels of immune checkpoint markers in lung CD4^+^ and CD8^+^ T cells were lower in *Ahr*^*Lyz-KO*^ mice (Fig. 8E, F, Supplementary Fig. 5F, G), whereas the expression levels of IFN-γ and TNF-α were greater (Fig. 8G, H, Supplementary Fig. 5H, I). These results suggest that the Kyn-AhR pathway influences the function of effector T cells. Next, we investigated the role of the Kyn-AhR pathway in the proliferation of Mtb and pathology in the lungs of mice. Therefore, we injected Kyn into Mtb-infected mice to investigate the effects of the tryptophan metabolite Kyn on disease burden and lung pathology in a mouse model. In wild-type (WT) mice, Kyn treatment significantly increased the bacterial burden in the lung and spleen (Fig. 8I, J). Colony forming unit (CFU) assays and acid-fast staining indicated that Ahr silencing enhanced the host immune response against Mtb, resulting in a reduced lung bacterial burden (Fig. 7K, L). These findings suggest that Kyn-Ahr pathway inhibition alleviates the disease burden in a mouse model of tuberculosis.

## Discussion

In this study, we demonstrated that Mtb promotes the expression of the tryptophan-metabolizing enzyme IDO1 in inflammatory macrophages and enhances the Kyn-AhR pathway to upregulate SOCS3 expression, thereby regulating the STAT1-CXCL9/10 signaling pathway (Supplementary Fig. 6). This affects T-cell infiltration and functionality in the lungs of mice infected with tuberculosis. More importantly, we discovered that this process might be an inherent metabolic-dependent negative feedback mechanism designed to suppress an overly activated STAT1‒CXCL9/10 pathway to prevent autoimmune damage. However, Mtb can exploit this process by inducing metabolic reprogramming in inflammatory macrophages at the site of lung infection, which abnormally enhances this feedback mechanism. This ultimately leads to Mtb evasion of interferon-dependent immune surveillance. The identification of this mechanism reveals one of the specific ways in which Mtb manipulates T-cell delay in reaching the site of infection and addresses the previous research gap concerning the immunosuppressive effects of AhR on the host.

During the process of tuberculosis infection, metabolic changes at the host site significantly impact the immune response [36]. However, the specific mechanisms by which Mtb interferes with host metabolism are not yet fully understood. Tryptophan metabolism plays a complex role in the interactions between hosts and microbes [37]. It can be a component of the host’s defense mechanisms and may also be exploited by pathogens to evade immune responses. Host macrophages consume tryptophan through the enzyme IDO1, a strategy intended to limit the virulence of pathogens that depend on exogenous tryptophan for survival, such as certain parasites, *Chlamydia trachomatis* and *Streptococcus pyogenes* [38, 39]. In contrast, Mtb has evolved the ability to synthesize tryptophan autonomously. This adaptation allows it to withstand the local depletion of tryptophan caused by the host’s increased expression of IDO1 during infection [40]. This enables the Mtb to persist despite the host’s defensive strategies aimed at limiting pathogen survival through nutrient restriction. Under physiological conditions, proinflammatory macrophages secrete cytokines and express IDO1 to maintain immune balance and prevent excessive inflammatory responses. However, we found that Mtb enhances the expression of IDO1 by stabilizing *IDO1* mRNA in inflammatory macrophages, which promotes tryptophan metabolism within the Mtb infection site. RNA-binding proteins (RBPs) can protect RNA molecules from degradation or promote the degradation of specific RNA molecules, thus regulating RNA stability and intracellular concentration. In a tumor model, ARID5A, an RNA-binding protein, acts as an mRNA stabilizer to promote IDO1 expression [23, 41]. Whether Mtb stabilizes IDO1 by regulating a specific RBP remains to be further explored in the future.

Previous studies have shown that IDO1 is expressed predominantly in macrophages and dendritic cells (DCs) within tuberculosis-infected lungs, with increased enrichment of macrophages [42]. This could be attributed to the distinct roles played by macrophages and DCs during Mtb infection. After Mtb infection, most macrophages remain in the lungs, where they secrete proinflammatory cytokines and chemokines to induce inflammatory responses and recruit immune cells. In contrast, immature DCs in the lungs recognize and phagocytose Mtb or specific antigens and then migrate to lymph nodes, where mature DCs participate in inducing T-cell-mediated immune responses against bacteria [43]. T cells are subsequently recruited to the site of lung infection, where cytokine crosstalk between T cells and macrophages becomes critical to the outcome of Mtb infection.

Kyn promotes DC adoption of a tolerogenic phenotype characterized by reduced expression of costimulatory molecules and increased expression of immunosuppressive molecules such as IL-10 and TGF-β, which further facilitates the differentiation and expansion of Tregs [44, 45].

In lymph nodes, an abundant accumulation of Mtb-specific Tregs can delay the activation and migration of effector T cells to the lungs [1]. Additionally, activation of AhR can directly alter the differentiation and intrinsic functions of inflammatory dendritic cells through a kyn-dependent mechanism without impairing their ability to present antigens to T cells or activate antigen-specific T cells in vivo [46]. AhR knockout can reduce the proliferation of Tregs [47]. In tuberculosis patients, overactivation of the Kyn-AhR pathway may increase the number or proportion of Tregs in the lymph nodes or lungs, which suppresses the infiltration and function of effector T cells in the lungs. Additionally, Tregs may induce the polarization of M1 inflammatory macrophages to an alternatively activated (M2-like) state, thereby suppressing the immune response [48]. These possibilities need further validation and in-depth research in the future.

Previous studies have shown that the use of IDO1 inhibitors can improve the distribution of T cells within tuberculosis granulomas in macaques, promoting increased infiltration of T cells into granulomas and thereby enhancing the effectiveness of bacterial eradication [21]. Although the exact mechanisms behind this phenomenon have not been fully elucidated, one possible explanation is that inhibition of IDO1 accelerates the migration of T cells to the lungs early in infection, thereby strengthening the host’s immune control over Mtb. In this study, we found that the expression levels of IDO1 in lung tissues from tuberculosis patients were negatively correlated with T-cell infiltration.

In the process of tryptophan metabolism, Kyn, catalyzed by IDO1, acts as an endogenous ligand that can activate AhR. The activation of AhR allows it to enter the nucleus and perform its transcriptional functions, thus playing a crucial role in the regulation of immune responses [49]. This interaction highlights the importance of AhR as a mediator in immune modulation, linking metabolic pathways to immune system regulation [50, 51]. AhR activity is associated with the pathogenesis of various diseases, including inflammatory diseases [52], neurological disorders [53] and cancer [54], making it an attractive target for the development of novel prevention and treatment strategies. In the field of tuberculosis, researchers have identified a significant role for AhR in the death of macrophages infected with Mtb via the use of CRISPR interference screening technology [55]. Additionally, AhR can accelerate the metabolism of the antituberculosis drug rifabutin, which may affect drug efficacy [56]. Given these findings, AhR antagonists have shown potential as a host-directed therapeutic strategy in animal models and may represent a new therapeutic approach.

The JAK-STAT1 pathway is an important signaling route in inflammatory macrophages for the secretion of cytokines that promote inflammation. Specifically, the conserved tyrosine and serine residues on the C-terminal domain of STAT1 are phosphorylated by JAK kinases and mitogen-activated protein kinases, respectively, which activate STAT1 [57]. This activation enables STAT1 to dimerize and translocate to the nucleus, where it regulates the expression of downstream chemokines, including CXCL9 and CXCL10. These chemokines play critical roles in recruiting immune cells to sites of inflammation or infection, thereby facilitating an effective immune response [58]. Within the context of inflammatory responses, the activation of AhR can regulate the expression of various genes, including members of the SOCS family [59]. SOCS family proteins interact with JAK kinases to inhibit the JAK-STAT signaling pathway, providing a negative feedback mechanism that prevents excessive activation of cytokine signaling and inflammation. In this study, qPCR screening revealed that AhR can upregulate the expression of SOCS3, a member of the SOCS family. Furthermore, chromatin immunoprecipitation assays and CUT&Tag-seq confirmed that AhR can directly bind to the promoter region of SOCS3. Thus, by increasing the expression of SOCS3, AhR inhibits the activity of the JAK-STAT1 signaling pathway, subsequently reducing the levels of CXCL9 and CXCL10. These findings indicate that AhR plays a significant role in modulating the adaptive immune response to tuberculosis.

The response of effector T cells to pulmonary Mtb infection is closely related to long-term protection [60, 61]. The delayed arrival of T cells to the lungs extends the initial phase of unrestricted bacterial replication. When effector T cells eventually reach the infection site, bacteria can induce a multilayered immunosuppressive microenvironment, such as the induction of myeloid-derived suppressor cells (MDSCs) [62], high levels of immunosuppressive cytokines [63], and increased expression of immune checkpoint molecules such as PD-L1 and CD39 on macrophages [64], limiting the ability of T cells to control or clear Mtb at the site of lung infection, thereby inducing dysfunction in effector T cells. In our study, we found that administering Kyn to mice not only significantly reduced T-cell infiltration in the lungs but also led to T-cell dysfunction. In contrast, in mice lacking the AhR in macrophages, we observed a significant increase in T-cell infiltration, along with increased cytokine secretion by CD4^+^ and CD8^+^ T cells, ultimately promoting an adaptive immune response against tuberculosis and reducing the bacterial load.

Given that the delay in the T-cell response is a major barrier to curing Mtb infection and one of the bottlenecks in the development of new tuberculosis vaccines, our findings provide important theoretical support for the development of host-directed therapeutic strategies that target AhR. Inhibiting AhR activity could help accelerate the immune response of T cells, thereby enhancing the host’s control over Mtb infection and opening new avenues for the treatment and vaccine development of tuberculosis.

## Methods

### Cell culture and Mtb infection

The THP-1 cells used in this study were obtained from ATCC and cultured in RPMI 1640 medium (Thermo Fisher Scientific, USA) supplemented with 10% fetal bovine serum (FBS) (Gibco, USA) and 1% penicillin‒streptomycin solution (Caisson, USA). The THP-1 cells used in the laboratory were subjected to examination for mycoplasma contamination and authenticated. THP-1 cells were differentiated with PMA (0.1 μM) beginning 36 h before transfection or other treatments. Once the cells were adherent, they were transferred to PMA-free media to obtain resting macrophages. The cells were pretreated for 24 h with 20 ng/mL IFN-γ to generate inflammatory macrophages before Mtb infection (MOI = 5). Four hours after Mtb infection, the infected cells were washed three times with 1× PBS and incubated again with fresh RPMI 1640 medium.

Mouse bone marrow-derived macrophages (BMDMs) were isolated from 6∼8-week-old C57BL/6 J mice and cultured in DMEM (Thermo Fisher Scientific, USA) supplemented with 10% FBS, 1% penicillin‒streptomycin solution, and 10 ng/mL murine macrophage colony‒stimulating factor (Pepro Tech, USA) for 4–6 days. All the cell lines used in the laboratory were cultured in a humidified incubator at 37 °C with 5% CO_2_ and were subjected to examination for mycoplasma contamination and authenticated. The cells were pretreated for 24 h with 20 ng/mL IFN-γ before Mtb infection (MOI = 5) for another indication. Four hours after Mtb infection, the infected cells were washed three times with 1× PBS and incubated again with fresh DMEM.

### Mice

The *Ahr*^flox/flox^ mice and *Lyz2*-cre mice were a gift from the Institute of Basic Medical Sciences, Chinese Academy of Medical Sciences, and were crossed to generate *Ahr*^*lyz*-KO^ mice. Six– to eight-week-old mice that were matched for age and sex were used and randomly allocated to experimental groups. The mice were housed under a 12/12 h light/dark cycle at 22–26 °C under specific pathogen-free (SPF) conditions and provided sterile pellet food and water *ad libitum*. The study is compliant with all of the relevant ethical regulations regarding animal research.

### Mouse infection

The mice were challenged by aerosol exposure to Mtb H37Rv via an inhalation device (Glas-Col) calibrated to deliver 100 CFUs of Mtb H37Rv. After infection, PBS or Kyn (20 mg/kg) was injected intraperitoneally into C57BL/6 J mice daily. After 3 or 6 weeks of infection, the lungs and spleens were harvested. Some lung samples were fixed in 4% paraformaldehyde and embedded in paraffin, after which sections were cut for acid‒fast staining and immunohistochemistry. Other samples were homogenized with a FastPrep-24 System (MP Biomedicals, USA) for qPCR analysis and CFU counting. The tissue slices were collected with a Leica CS2 and analyzed with Slide Viewer and ImageJ software. The sample size was based on empirical data from pilot experiments.

### Quantitative Polymerase Chain Reaction (qPCR)

For the purification of total RNA from cultured cells, the cells were collected, and total RNA was extracted via TRIzol (Thermo Fisher Scientific, USA). For the purification of total RNA from formalin-fixed, paraffin-embedded (FFPE) samples, the protocol of the RecoverAll™ Total Nucleic Acid Isolation Kit for FFPE samples was followed. Total RNA was extracted from paraffin-embedded lung tissue sections from tuberculosis patients. The process began with deparaffinization using xylene and ethanol to remove the paraffin, followed by protease digestion at specified temperatures (50 °C/80 °C for RNA) to break down protein‒nucleic acid crosslinks. Nucleic acids were then isolated via a glass-fiber filter method, which included an integrated nuclease treatment to eliminate contaminants. The nucleic acids were subsequently purified and eluted in water or low-salt buffer. The total RNA was then reverse transcribed into cDNA via reverse transcriptase (TAKARA, Japan) and subjected to qPCR analysis with a QuantiNova SYBR PCR Mix Kit (QIAGEN, Germany) on an ABI 7500 system (Applied Biosystems, USA). The quantitative expression of the targeted gene was standardized against that of GAPDH. All the qPCR primers used can be found in Supplementary Table 2.

### Western blot

The cells were harvested and lysed in RIPA lysis buffer (Servicebio, China) supplemented with a protease and phosphatase inhibitor cocktail (Cell Signaling Technology, USA). Protein concentrations were determined with a BCA kit (Thermo Fisher Scientific, USA). All protein samples were subsequently separated via SDS‒PAGE, after which the target proteins were transferred onto polyvinylidene fluoride membranes. The NC membranes were blocked in 5% BSA and probed overnight with the indicated antibodies at 4 °C (Supplementary Table 1). The addition of secondary antibodies conjugated to horseradish peroxidase was followed by enhanced chemiluminescence (Thermo Fisher Scientific, USA). The protein bands were visualized via chemiluminescence according to the manufacturers’ instructions.

### Immunofluorescence

The cells were plated on glass coverslips in 24-well plates, fixed in 4% paraformaldehyde and permeabilized with 0.2% Triton X-100 (Sigma‒Aldrich, Germany). Fixed cells were blocked with 3% BSA in PBST (PBS with 0.2% Triton X-100) for 1 h prior to incubation with primary antibodies overnight at 4 °C. The cells were then stained with secondary antibodies (Alexa Fluor 488, Invitrogen, USA), and the nuclei were stained with DAPI (Beyotime, China) and mounted for confocal analysis under a Zeiss LSM980 confocal laser microscope. The intensity of immunofluorescence was analyzed by ImageJ software.

### Immunohistochemistry

After tissue collection and fixation with 4% paraformaldehyde, the sectioned tissue was boiled in citrate buffer (pH 6.0) for 10 min, washed three times, and blocked with 3% BSA in PBST (0.2% Triton X-100), followed by incubation with primary antibodies at 4 °C overnight. The slides were then incubated sequentially with the secondary antibody for 1 h at room temperature. After development in Tyramide Signal Amplification Plus Working Solution for 5 min, the slides were counterstained with hematoxylin and mounted for image analysis.

### Acid-fast staining

Mouse lung tissue sections were first flooded with carbol fuchsin, a lipid-soluble dye, and heated for 5 min. After cooling, the slides were washed and decolorized with acid‒alcohol. The slides were then counterstained with methylene blue, followed by rinsing, drying, and sealing. Finally, the prepared sections were observed under a 100× oil immersion microscope. Image capture was conducted via DX12 (3DHISTECH, Germany).

### Flow cytometry

After isolation, the lungs were digested with 5 g/L collagenase D (Roche, Switzerland) and 2 × 10^6^ units/L DNase (Roche, Switzerland) for 30 min at 37 °C. Single-cell suspensions from mouse lungs were prepared. Human peripheral blood samples were obtained from Beijing Chest Hospital, Capital Medical University. Single-cell suspensions from mouse blood samples or human blood samples were prepared. All samples were resuspended in PBS containing 1% FBS and stained with live/dead dye (BioLegend, USA) for 15 min at room temperature before they were stained with different antibodies at the indicated times. For the staining of intracellular markers, the cells were fixed and permeabilized after surface stain incubation. The data were analyzed on a BD Aria III cytometer and processed via FlowJo v10.5.3 software (BD).

### ELISA

Chemokine secretion was quantified by using corresponding ELISA kits according to the manufacturer’s protocol.

### RNAi

THP-1 cells and BMDMs were transfected with siRNAs via RNAiMAX (Thermo Fisher Scientific, USA) transfection reagent for 48 h before other treatments. Synthetic small interfering RNA (siRNA) oligonucleotides were synthesized by Sigma or Life Technologies. The knockdown efficiency was assessed 48 h after transfection via western blotting. The sequences of the siRNAs can be found in Supplementary Table 2.

### Cell transfection

Recombinant plasmids encoding murine or human *IDO1* and *AhR* were constructed via PCR-based amplification from the cDNA of Sino Biological. For jetPRIME-mediated transfection in a 12-well format, 1 μg of plasmid was diluted with 100 μL of jetPRIME® buffer and mixed well before adding 2 μL of jetPRIME® reagent. This mixture was incubated at room temperature for 10 min and added to the cells in serum-containing medium. The medium was changed after 12 h of transfection, and the cells were grown further for 12 h before being processed. The plasmids used can be found in Supplementary Table 2.

### Transwell assay

The CD4^+^ or CD8^+^ T lymphocytes were sorted via a T-cell isolation kit (Miltenyi Biotec, Germany) and then stimulated with anti-CD3/CD28 Dynabeads (Thermo Fisher Scientific, USA) and 100 U/mL human IL-2 (PeproTech, USA) for 96 h. T cells in 100 μL of complete media were loaded into the top chamber of Transwell inserts (5.0 μm pore size; Corning). The bottom well was filled with RPMI 1640 medium or CM derived from macrophages subjected to different treatment conditions. To block CXCL9/CXCL10, T cells were preincubated with 10 μg/mL anti-CXCL9/CXCL10 (BioLegend, USA). for 30 min prior to loading into the top chamber. The plates were incubated at 37 °C overnight, the contents of the lower chamber were collected, and the viable T lymphocytes were counted with trypan blue.

### ChIP‒qPCR

ChIP‒qPCR was performed via the MAGnity Chromatin Immunoprecipitation System (Invitrogen, USA) according to the manufacturer’s instructions. In brief, THP-1 cells were cross-linked, and chromatin was extracted and sheared to an average fragment size of 200–1000 bp. The samples were immunoprecipitated with an anti-AhR antibody (GeneTex, USA). The immunoprecipitated DNA fragments were then analyzed via qPCR on an ABI 7500 qPCR System. The sequences of primers used for ChIP‒qPCR can be found in Supplementary Table 2.

### Luciferase assays

THP-1 cells were transfected with 100 ng of the Renilla luciferase plasmid pRL-SV40, 1 μg of the firefly luciferase plasmid pGL3-*SOCS3* and 1 μg of the pCMV6–*AhR* plasmid for 12 h. Then, these cells were treated with or without Kyn (100 μM) for 24 h. Cell lysates were analyzed via the Dual Luciferase Reporter Assay (Promega, USA) on a GloMax Multi Plus (Promega, USA). Firefly luciferase activity was normalized to that of Renilla luciferase.

### mRNA decay assay

The mRNA decay assay was conducted as previously described [65]. Briefly, THP-1 cells were pretreated with IFN-γ (20 ng/mL) for 24 h, infected with Mtb at an MOI of 5 for 12 h, or left uninfected as a control. The cells were treated with actinomycin D (10 μg/mL) to halt transcription. RNA was extracted from these cells and reverse transcribed into cDNA via reverse transcriptase (TAKARA, Japan). Quantitative PCR (qPCR) was then performed via the QuantiNova SYBR PCR Mix Kit (QIAGEN, Germany) on an ABI 7500 system (Applied Biosystems, USA).

### CUT&Tag assay

The CUT&Tag assay was conducted following the instructions of the Hyperactive Universal CUT&Tag Assay Kit for Illumina Pro (catalog number TD904; Vazyme, Nanjing, China). For the preparation of the indexed DNA libraries, the TruePrep Index Kit V4 (catalog number TD202; Vazyme) was utilized. The libraries were then sequenced via an Illumina NovaSeq 6000 (PE150) platform. The quality of the NSG data was assessed via FastQC. The associated CUT&Tag datasets are accessible in the Science Data Bank (10.57760/sciencedb.13507).

### LC‒MS analysis of serum Kyn levels

Serum Kyn levels in tuberculosis patients and healthy individuals were determined via an LC‒MS system according to previous methods (https://www.nature.com/articles/s41598-023-39774-3#Sec4). An Agilent 6495 triple quadrupole LC–MS system and a C18 chromatographic column (Thermo Fisher Scientific, 100 mm × 2.1 mm, 1.7 μm) were utilized for analysis. Gradient elution was employed for LC separation, with 0.1% formic acid as solvent A and pure acetonitrile as solvent B. The flow rate was set at 0.3 ml min^−1^, and the injection volume was 10 μl. The gradient program was as follows: 0–1 min, 95% A; 1–6 min, 95% A to 5% A; 6–7 min, 5% A; 7–7.2 min, 5% A to 95% A; and 7.2–11 min, 95% A. Each sample had a total run time of 11 min, and data were collected via data acquisition.

### Ethical approval

All study procedures involving human blood samples were approved by the Ethics Committee of Beijing Chest Hospital, which is affiliated with Capital Medical University (approval No. YJS-2022-03). TB patient lung tissue sections and corresponding blood samples were acquired from the Fifth People’s Hospital of Suzhou from sixteen patients who underwent pretreatment diagnostic biopsy (SZWY2023-013). Patients with HIV coinfection or tumors or other pathogens were excluded. Informed consent was obtained from all participants in accordance with the Declaration of Helsinki. Written informed consent was obtained from each individual. The clinical features of the patients are listed in Supplementary Tables 3–6. All the animal procedures were approved by the Ethics Committee of the Experimental Animal Care of Beijing Chest Hospital, Capital Medical University (Approval No. 2023-006). All experimental protocols for the experiments on *M. tuberculosis* were approved by the Biosafety Committee of Beijing Chest Hospital and conducted in accordance with national biosafety guidelines in China.

### Statistical analysis

All experiments were performed with at least three biological replicates with similar results. The analysis was conducted via GraphPad 8.0 software. The results are expressed as the means ± SDs as indicated, and the data were analyzed via two-tailed Student’s *t* test or one-way analysis of variance (ANOVA). *p* < 0.05 was considered statistically significant. *p < 0.05, **p < 0.01, ***p < 0.001, ns, not significant.

## Supplementary information

Supplementary Information

Uncropped images of all western blot
