# Supplementary material for: Kynurenine-AhR reduces T-cell infiltration and induces a delayed T-cell immune response by suppressing the STAT1-CXCL9/CXCL10 axis in tuberculosis
Source: Cell Mol Immunol. 2024 Oct 22;21(12):1426–40. doi: 10.1038/s41423-024-01230-1 (PMC11607402; doi:10.1038/s41423-024-01230-1)
Supplement: Supplementary file 1 — Supplementary Information [file 41423_2024_1230_MOESM1_ESM.docx]

**Supplementary Materials for**

**Kynurenine-AhR reduces T cell infiltration and induces a delayed T cell immune response by suppressing the STAT1-CXCL9/CXCL10 axis in tuberculosis**

Xin Liu *et al*

Corresponding author: Yu Pang (pangyupound@163.com), Jinfeng Yuan (yuanjinfeng0920@163.com)

**The PDF file includes:**

Figs. S1 to S6

Tables S1 to S6


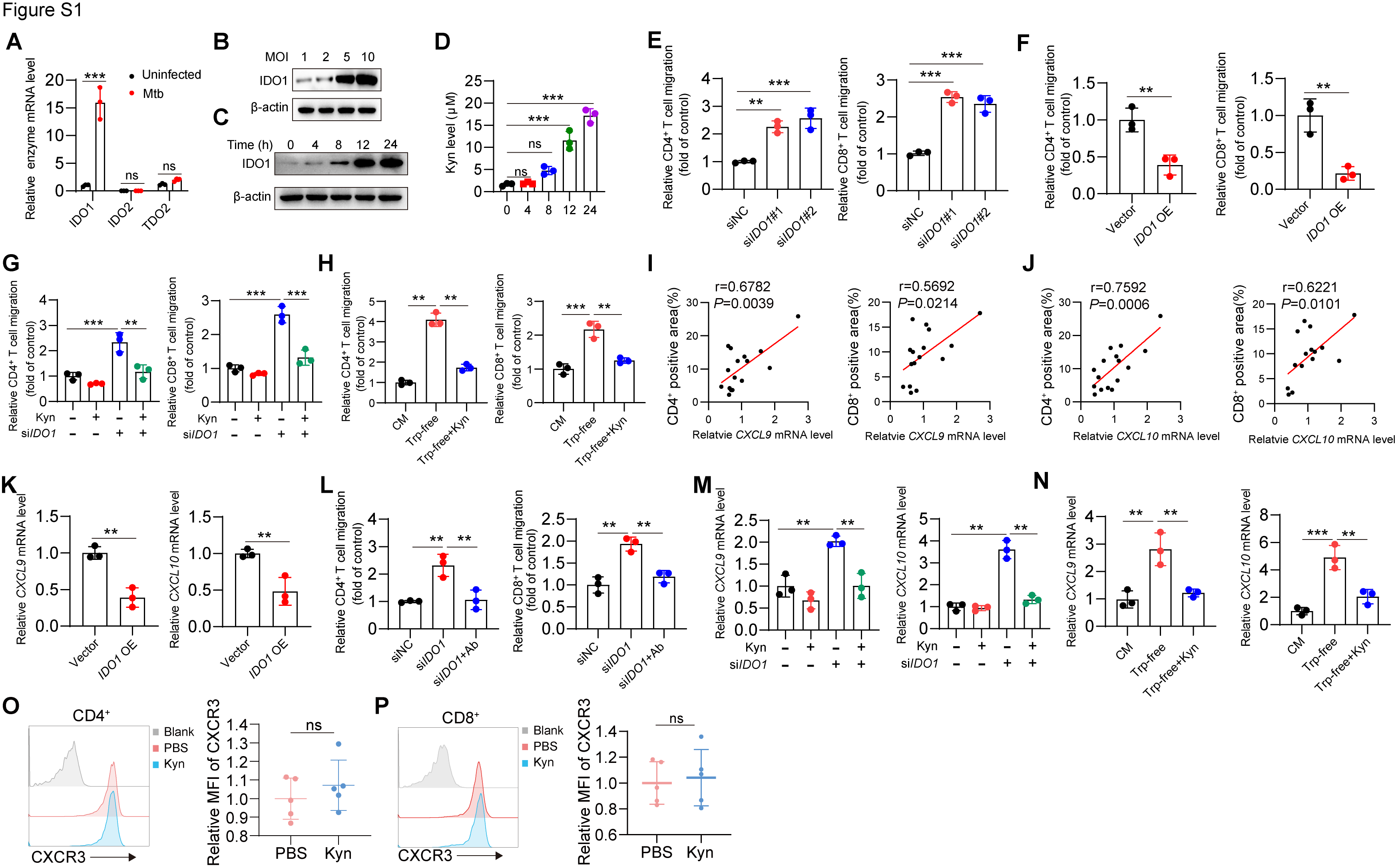


**Supplemental Fig.1. Mtb regulates T cell infiltration by activating IDO1-Kyn pathway.**

**(A**) qPCR analysis of indicated mRNA in BMDMs pretreated with IFN-γ (20 ng/mL) for 24 h was infected with or without Mtb (MOI=5) for 24 h. **(B)** BMDMs were pretreated with IFN-γ (20 ng/mL) for 24 h, then infected with different MOI of Mtb. Indicated antibodies were measured by western blot analysis. **(C)** BMDMs were pretreated with IFN-γ (20 ng/mL) for 24 h, then infected with Mtb for the indicated hours. Indicated antibodies were measured by western blot analysis. **(D)** LC-MS detection of Kyn levels in BMDMs pretreated with IFN-γ (20 ng/mL) for 24 h was infected with Mtb for the indicated hours. **(E)** BMDMs were transfected with NC or *IDO1* siRNA for 24 h, followed by IFN-γ (20 ng/mL) treatment for 24 h. The CD4^+^ and CD8^+^ T cells migration was measured by transwell migration assay. **(F)** BMDMs were transfected with or without IDO1 by jetPRIME, followed by IFN-γ (20 ng/mL) treatment for 24 h. Then the CD4^+^ and CD8^+^ T cells migration was measured by transwell migration assay. **(G)** The same as **(E)**, except that the cells were treated with or without Kyn (100 μM) for 24 h. The CD4^+^ and CD8^+^ T cells migration was measured by transwell migration assay. **(H)** THP-1 cells were cultured in complete medium and Trp-free medium with or without Kyn (100 μM) for 24 h. The CD4^+^ and CD8^+^ T cells migration was measured by transwell migration assay. **(I)** The correlation between the mRNA expression of CXCL9, extracted from lung tissue sections of tuberculosis patients, and the CD4+ or CD8+ positive areas stained by immunohistochemistry in the lungs of these patients. Statistical significance was measured by Spearman’s correlation test. **(J)** The correlation between the mRNA expression of CXCL10, extracted from lung tissue sections of tuberculosis patients, and the CD4^+^ or CD8^+^ positive areas stained by immunohistochemistry in the lungs of these patients. Statistical significance was measured by Spearman’s correlation test.**(K)** The same as **(F)**, then qPCR of indicated genes was performed. **(L)** The same as **(E)**, except that the cells were treated with or without CXCL9 and CXCL10 neutralizing antibodies for 24 h. The CD4^+^ and CD8^+^ T cells infiltration was measured by transwell migration assay. **(M)** The same as **(G)**, then qPCR analysis of indicated mRNA was performed. **(N)** The same as **(H)**, then ELISA of CXCL9/10 was performed. (O-P) C57BL/6J mice Infected with the indicated Mtb strains by aerosol (~ 100 CFUs), then Mtb-infected C57BL/6J mice were treated with PBS or Kyn daily starting one day after infection for 3 weeks (n=5 mice per group). Then the lungs were harvested at 3 weeks. Single-cell suspensions of the lungs from the two treatment groups were stained with appropriate antibodies and analyzed using multicolor-flow cytometry. Flow cytometric analysis of the expression of CXCR3 in CD4^+^ T cell **(O)** and CD8^+^ T cell **(P)**. Data are presented as means ± SD. *p* values were calculated using one-way ANOVA [**(D)**, **(E)**, **(G)**, **(H)**, **(L)** to **(N)**] or unpaired two-tailed Student’s *t* test [**(A)**, **(F)**, **(K)**, **(O), (P)**]. ***p* < 0.01, ****p* < 0.001; ns, not significant (*p* > 0.05).


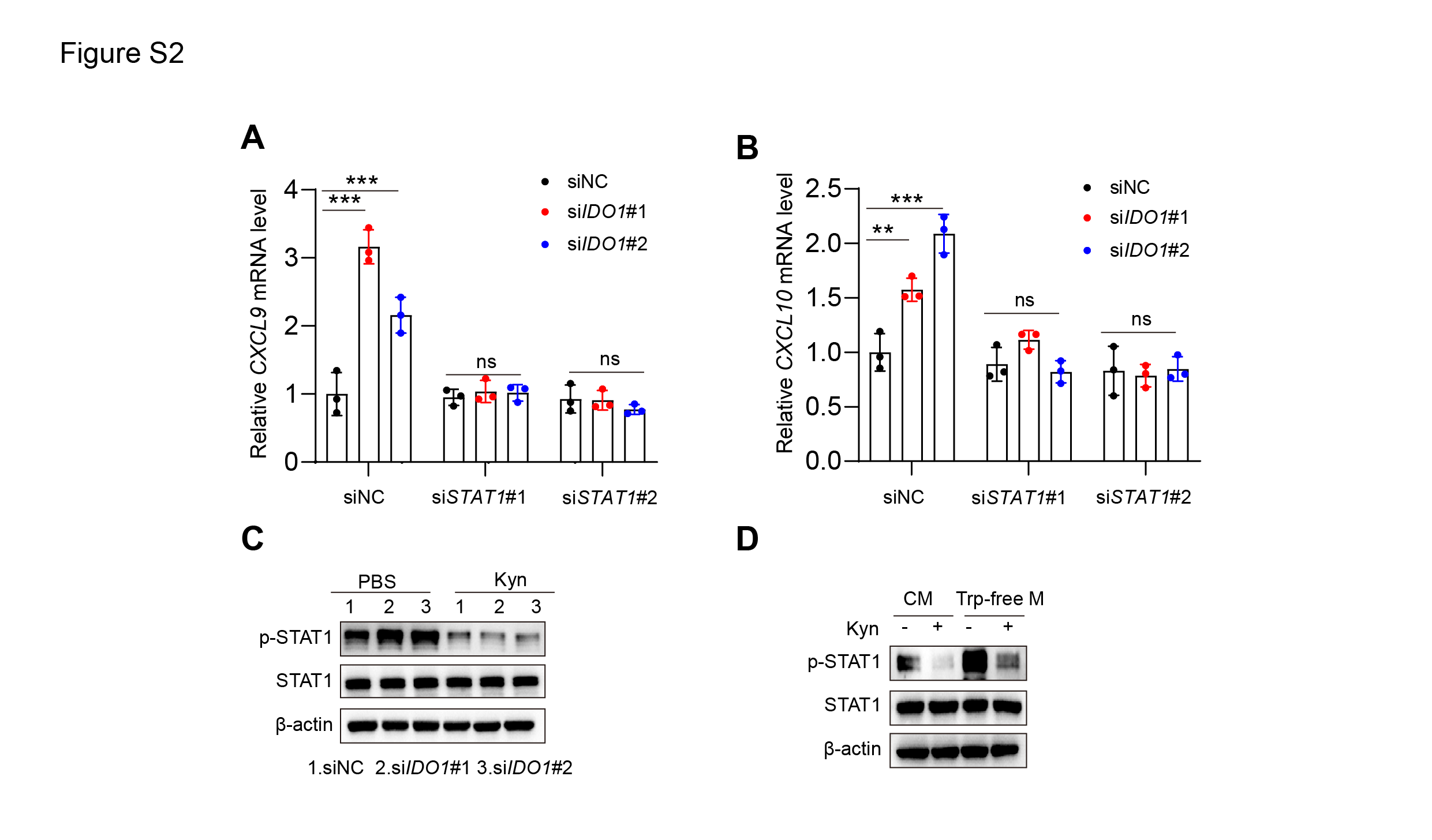


**Supplemental Fig.2. IDO1 supresses CXCL9/10 expression by inhibiting STAT1 pathway.**

**(A-B)** BMDMs were transfected with NC, *IDO1*, or cotransfected with *IDO1* and *STAT1* siRNA for 24 h, followed by IFN-γ (20 ng/mL) treatment for 24 h. Then qPCR of indicated genes was performed. **(C)** The same as **(A)**, except that the cells were treated with or without Kyn (100 μM) for 24 h. Then indicated antibodies were measured by western blot analysis. **(D)** BMDMs were cultured in a complete medium and Trp-free medium with or without Kyn (100 μM) for 24 h, then indicated antibodies were measured by western blot analysis. Data are presented as means ± SD. *p* values were calculated using one-way ANOVA. ***p* < 0.01, ****p* < 0.001; ns, not significant (*p* > 0.05).


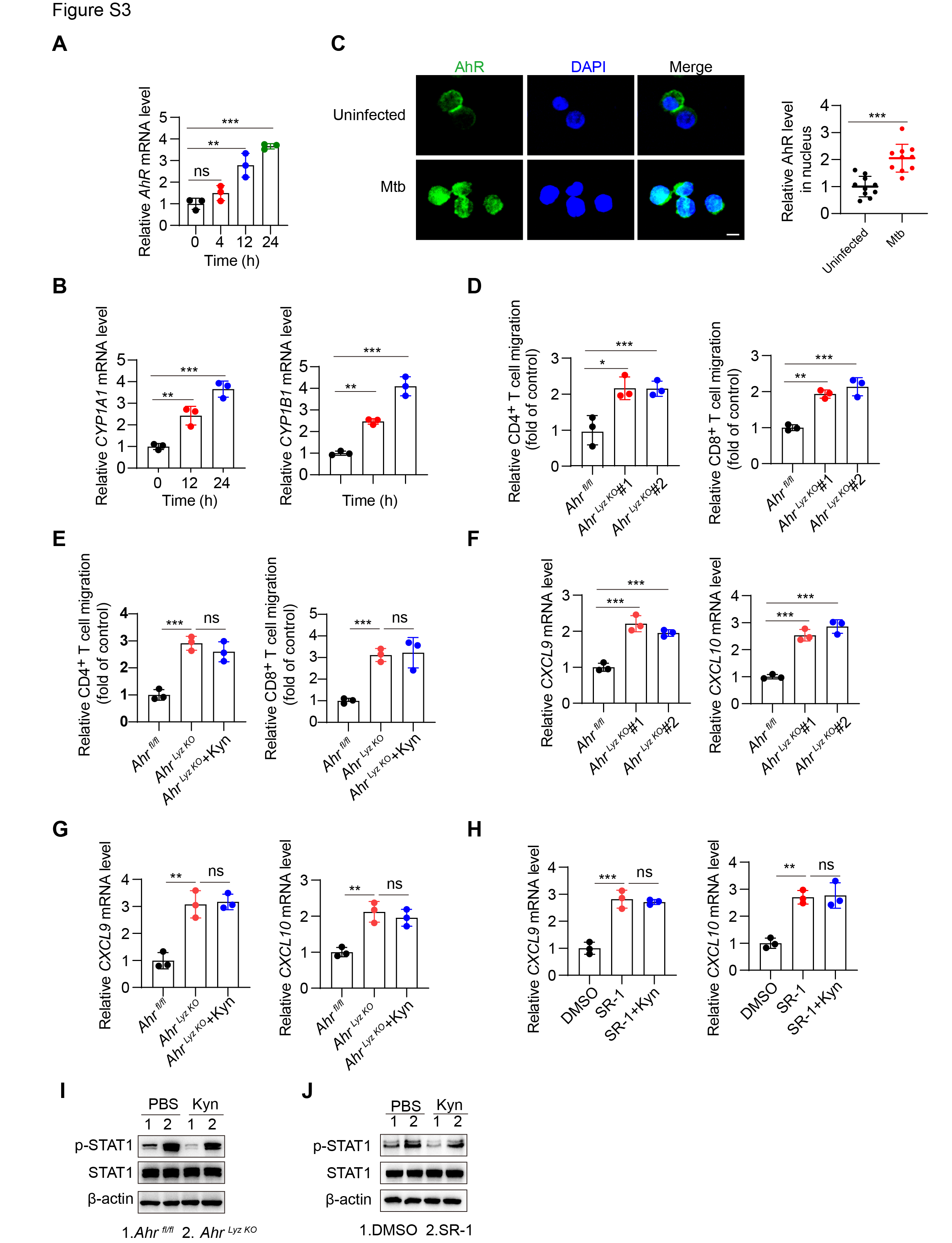


**Supplemental Fig.3.** **Mtb activates AhR to inhibit STAT1-CXCL9/10 pathway and T cell infiltration.**

**(A-B)** BMDMs were pretreated with IFN-γ (20 ng/mL) for 24 h, then infected with Mtb for the indicated hours. Then qPCR of indicated genes was performed. **(C)** The same as **(A)**, then the cells were stained for AhR and analyzed by confocal microscopy. Scale bars, 20 μm. **(D)** *Ahr^fl/fl^* mice and *Ahr^Lyz KO^* mouse BMDMs were pretreated with IFN-γ (20 ng/mL) for 24 h. Then the CD4^+^ and CD8^+^ T cells migration was measured by transwell migration assay. **(E)** The same as **(D)**, except that the cells were treated with or without Kyn (100 μM) for 24 h. Then the CD4^+^ and CD8^+^ T cells infiltration was measured by transwell migration assay. **(F)** The same as **(D)**, then qPCR of indicated genes was performed. **(G)** The same as **(D)**, except that the cells were treated with or without Kyn (100 μM) for 24 h. Then qPCR of indicated genes was performed. **(H**) BMDMs were pretreated with IFN-γ (20 ng/mL) for 24 h, followed by Kyn (100 μM) or Kyn combined with SR-1 (1 μM) for 24 h. Then qPCR of indicated genes was performed. **(I)** The same as **(D)**, except that the cells were treated with or without Kyn (100 μM) for 24 h. Then indicated antibodies were determined by western blot analysis. **(J)** BMDMs were pretreated with IFN-γ (20 ng/mL) for 24 h, followed by Kyn (100 μM) or Kyn combined with SR-1 (1 μM) for 24 h. Then indicated antibodies were determined by western blot analysis. Data are presented as means ± SD. *p* values were calculated using one-way ANOVA [**(A)**, **(B)**, **(D)** to **(H)**] or unpaired two-tailed Student’s *t* test **(C)**. **p* < 0.05, ***p* < 0.01, ****p* < 0.001; ns, not significant (*p* > 0.05).


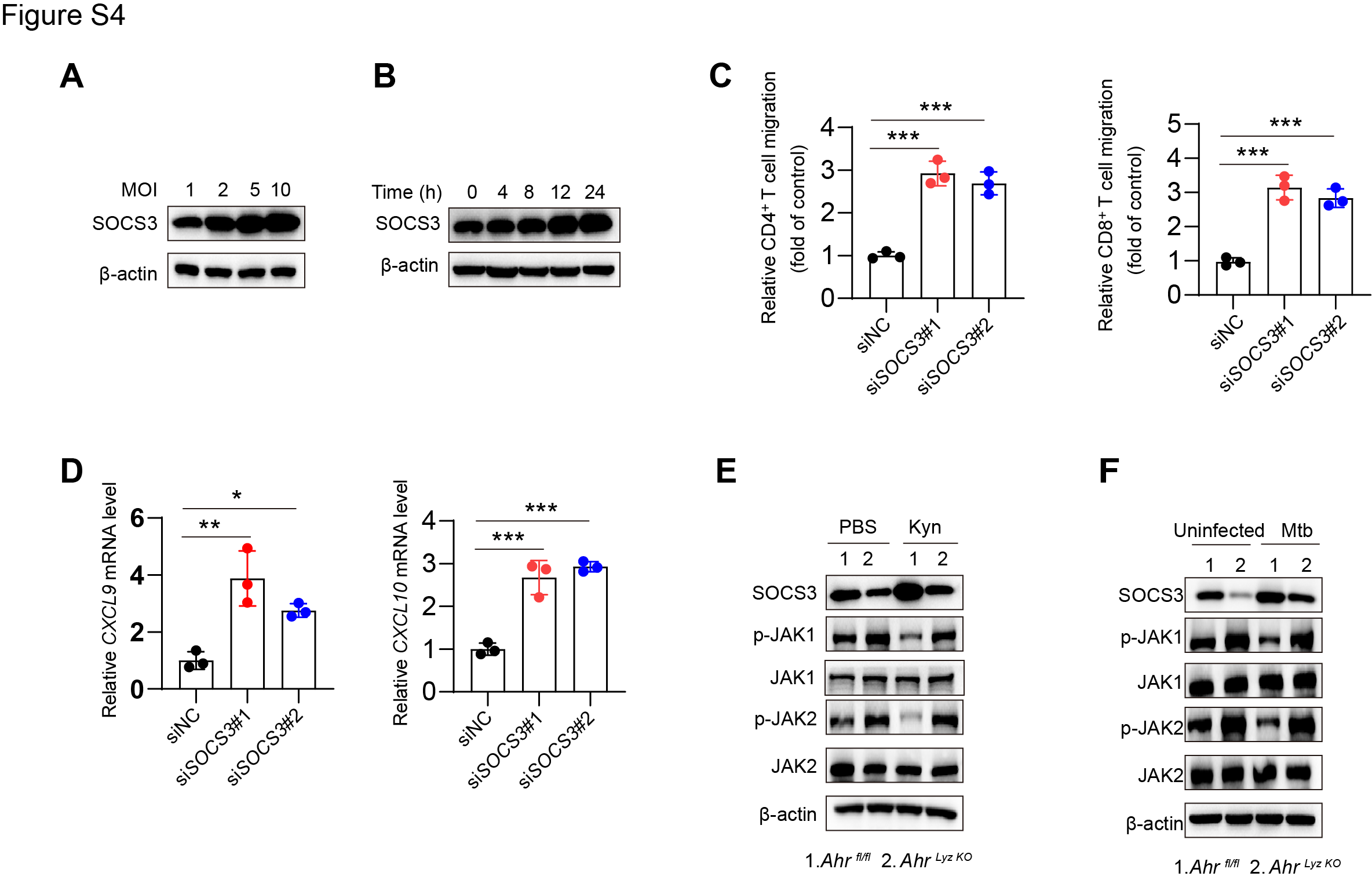


**Supplemental Fig.4. SOCS3 inhibits STAT1-CXCL9/10 pathway.**

**(A)** BMDMs were pretreated with IFN-γ (20 ng/mL) for 24 h, then infected with different MOI of Mtb. Indicated antibodies were measured by western blot analysis. **(B)** BMDMs were pretreated with IFN-γ (20 ng/mL) for 24 h, then infected with Mtb for the indicated hours. Indicated antibodies were measured by western blot analysis. **(C)** BMDMs were transfected with NC, *SOCS3* siRNA for 24 h, followed by IFN-γ (20 ng/mL) treatment for 24 h. Then the CD4^+^ and CD8^+^ T cells migration was measured by transwell migration assay. **(D)** The same as **(C)**, then qPCR of indicated genes was performed. **(E)** The same as **(C)**, except that the cells were treated with or without Kyn (100 μM) for 24 h. Then indicated antibodies were determined by western blot analysis. **(F)** The same as **(C)**, except that the cells were infected with or without Mtb (MOI=5) for 24 h. Then indicated antibodies were determined by western blot analysis. Data are presented as means ± SD. *p* values were calculated using one-way ANOVA. **p* < 0.05, ***p* < 0.01, ****p* < 0.001.


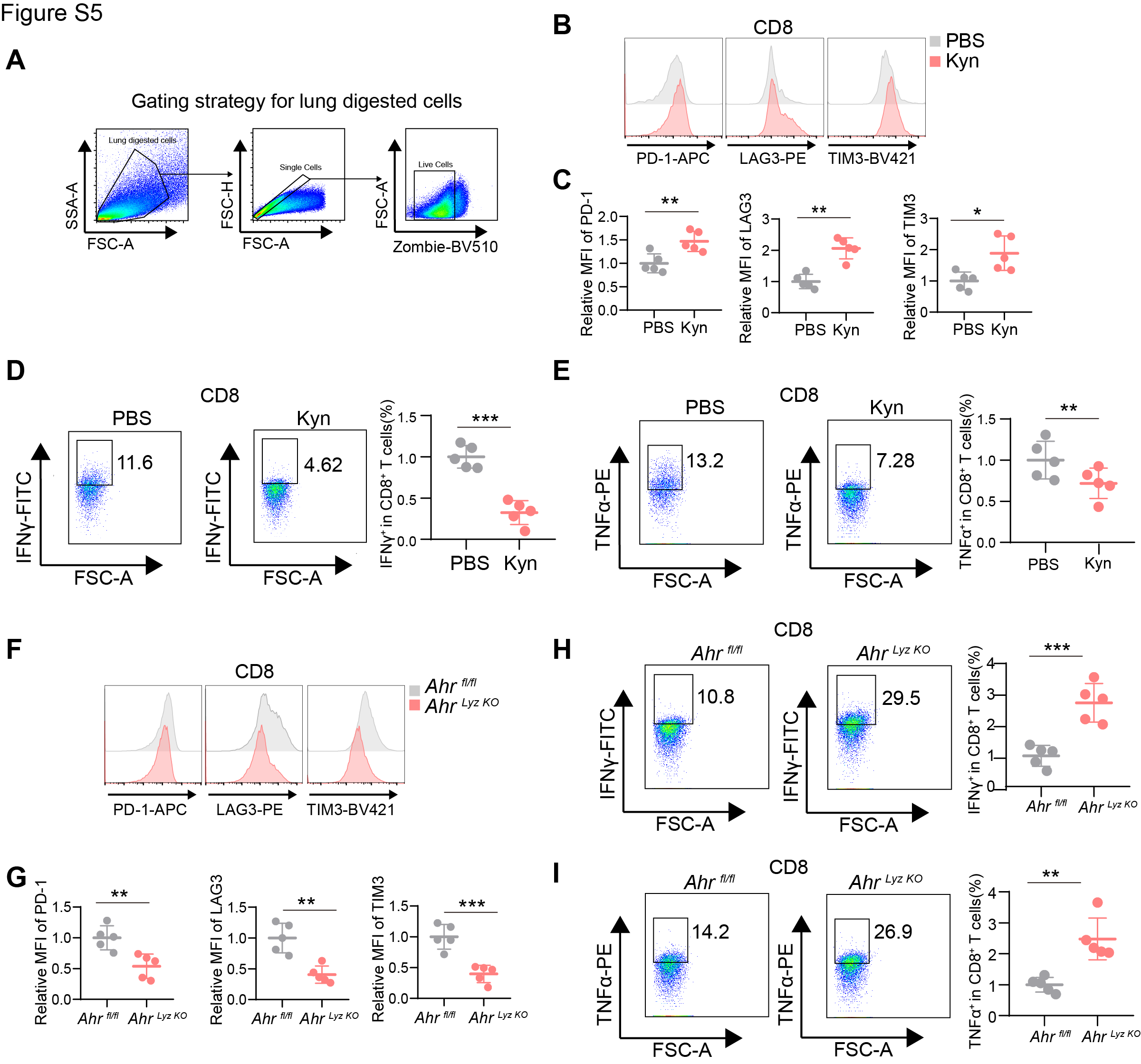


**Supplemental Fig.5 The Kyn–AhR pathway leads to CD8^+^ T cell dysfunction**

**(A-E)** Mtb-infected C57BL/6J mice (n = 5 mice per group) were treated with PBS or Kyn daily starting one day after infection. Mice were sacrificed after six weeks, and the lungs and spleens were harvested. Single-cell suspensions of the lungs from the two treatment groups were stained with appropriate antibodies and analyzed using multicolor-flow cytometry. **(A)** Gating strategies for flow cytometrical identification of T-cell subset. **(B-C)** We found differences in the frequencies or gMFI for PD-1, LAG3, TIM3 expression on activated CD8^+^ T cells. **(D)** IFN-γ expression on activated CD8^+^ T cells **(E)** TNF-α expression on activated CD8^+^ T cells. **(F-I)** Mtb-infected *Ahr^fl/fl^* and *Ahr^Lyz KO^* mice were sacrificed after six weeks, and the lungs were harvested (n = 5 mice per group). Single-cell suspensions of the lungs from the two treatment groups were stained with appropriate antibodies and analyzed using multicolor-flow cytometry. **(F-G)** We found differences in the frequencies or gMFI for PD-1, LAG3, TIM3 expression on activated CD8^+^ T cells. **(H)** IFN-γ expression on activated CD8^+^ T cells. **(I)** TNF-α expression on activated CD8^+^ T cells. gMFI was mostly used for low abundance cell surface markers and transcription factors. Data are presented as means ± SD. *p* values were calculated using two-tailed Student’s *t* test. **p* < 0.05, ***p* < 0.01, ****p* < 0.001.


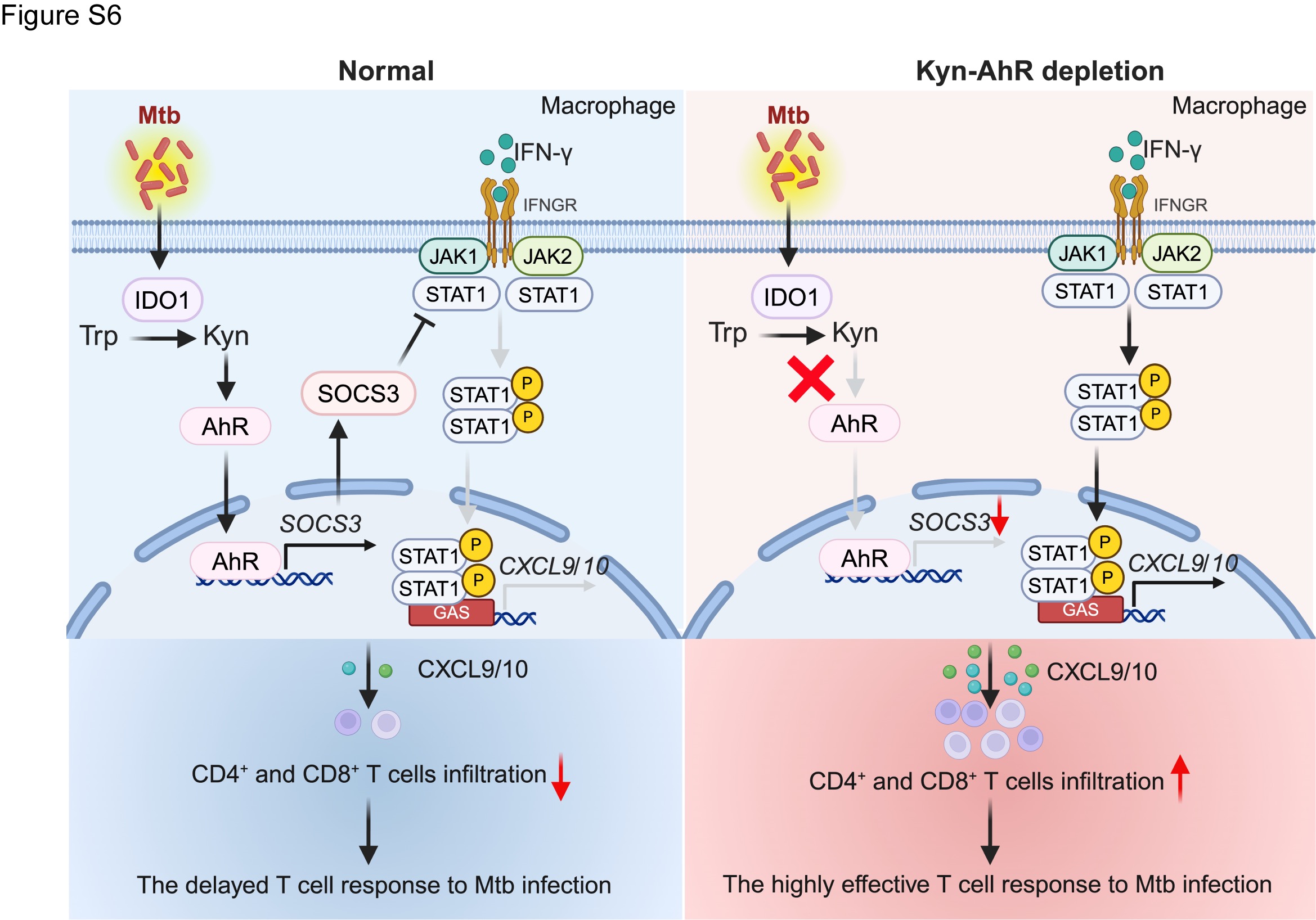


**Supplemental Fig.6 A proposed model showing the mechanism underlying Mtb-mediated delayed effector T cell migration to the lung**

Mtb promotes the expression of IDO1 on inflammatory macrophages, initiating downstream Kyn-AhR signaling to regulate the SCOS3-JAK-STAT1-CXCL9/10 signaling pathway, thereby reducing the migration of T cells to the site of infection and causing delayed adaptive immune responses.

**Table S1 Key Resources**

| **REAGENT or RESOURCE** | **SOURCE** | **IDENTIFIER** |
| --- | --- | --- |
| **Antibodies** |  |  |
| anti-STAT1 | Cell Signaling Technology | Cat# 9172; RRID: AB_2198289 |
| anti-JAK1 | Cell Signaling Technology | Cat# 5099; RRID: AB_2716281 |
| anti-JAK2 | Cell Signaling Technology | Cat# 3230; RRID: AB_2128522 |
| anti-Phospho-JAK1 | Cell Signaling Technology | Cat# 74129; RRID: AB_2799851 |
| anti-Phospho-JAK2 | Cell Signaling Technology | Cat# 8082; RRID: AB_10949104 |
| anti-Phospho-STAT1 | Cell Signaling Technology | Cat# 7649; RRID: AB_11220426 |
| anti-β-Actin | Cell Signaling Technology | Cat# 3700; RRID: AB_2242334 |
| anti-α-tubulin | Sigma-Aldrich | Cat# T5168; RRID: AB_477579 |
| anti-AhR | Cell Signaling Technology | Cat# 83200; RRID: AB_2800011 |
| anti-AhR | Genetex | Cat# GTX22769; RRID: AB_384839 |
| anti-IDO1 (human) | Abcam | Cat# ab211017; RRID: AB_2936946 |
| anti-IDO1 (mouse) | Abcam | Cat# ab311847 |
| APC/Cy7 anti-mouse CD8a | BioLegend | Cat# 100714; RRID: AB_312752 |
| PE/Cy7 anti-mouse CD4 | BioLegend | Cat# 100422; RRID: AB_312707 |
| PE anti-mouse TNF-α | BioLegend | Cat# 506306; RRID: AB_315427 |
| Brilliant Violet 711™ anti-mouse CD3 | BioLegend | Cat# 100241; RRID: AB_2563945 |
| FITC anti-mouse IFN-γ | BioLegend | Cat# 505806; RRID: AB_315400 |
| APC anti-mouse CD279 (PD-1) | BioLegend | Cat# 135209; RRID: AB_2251944 |
| PE anti-mouse CD223 (LAG-3) | BioLegend | Cat# 125207; RRID: AB_2133344 |
| FITC anti-mouse CD183(CXCR3) | BioLegend | Cat# 126536; RRID:AB_2566565 |
| Brilliant Violet 421™ anti-mouse CD366 (Tim-3) | BioLegend | Cat# 134019; RRID: AB_2814028 |
| anti-SOCS3 | Proteintech | Cat# 14025-1-AP; RRID: AB_10597854 |
| Goat Anti-Rabbit IgG H&L (HRP) | Jackson ImmunoResearch Labs | Cat# 111-035-003; RRID: AB_2313567 |
| Goat Anti-Mouse IgG H&L (HRP) | Jackson ImmunoResearch Labs | Cat# 115-035-003; RRID: AB_10015289 |
| Donkey anti-Mouse IgG (H+L) Highly Cross-Adsorbed Secondary Antibody, Alexa Fluor™ 488 | Invitrogen | Cat# A-21202 RRID: AB_141607 |
| Recombinant Human CXCL10 | BioLegend | Cat# 573504 |
| Recombinant Human CXCL9 | BioLegend | Cat# 578104 |
| Ultra-LEAF™ Purified anti-mouse CXCL9 | BioLegend | Cat# 942604 |
| Ultra-LEAF™ Purified anti-mouse CXCL10 | BioLegend | Cat#604454 |
| Bacterial and virus strains |  |  |
| H37Rv | ATCC | Cat# 27294 |
| **Chemicals, peptides, and recombinant proteins** |  |  |
| Cell Activation Cocktail (with Brefeldin A) | BioLegend | Cat# 423303 |
| Dynabeads™ Human T-Activator CD3/CD28 | Thermo Fisher Scientific | Cat# 11161D |
| UltraComp eBeads™ Compensation Beads | Thermo Fisher Scientific | Cat# 01-2222-42 |
| IC Fixation Buffer | Thermo Fisher Scientific | Cat# 00-8222-49 |
| Permeabilization Buffer | Thermo Fisher Scientific | Cat# 00-8333-56 |
| Trizol | Thermo Fisher Scientific | Cat# 15596 |
| Lipofectamine RNAiMAX | Thermo Fisher Scientific | Cat# 13778030 |
| Lipofectamine 2000 | Invitrogen | Cat#11668019 |
| Penicillin-Streptomycin Solution | Caisson Labs | Cat# PSL01-100ML |
| RPMI 1640 medium | Thermo Fisher Scientific | Cat# 11875093 |
| Trp-free RPMI 1640 medium | Boster | Cat# DZPYG0043 |
| DMEM medium | Thermo Fisher Scientific | Cat# 11965092 |
| Fetal Bovine Serum | Gibco | Cat# 10099141C |
| Recombinant Murine GM-CSF | PeproTech | Cat# 315-03 |
| Collagenase D | Roche | Cat# [11088858001](https://www.sigmaaldrich.cn/CN/zh/product/roche/colld-ro) |
| DNase | Roche | Cat# 10104159001 |
| RIPA lysis buffer | Servicebio | Cat# G2002-100ML |
| Pierce® Western Blot Signal Enhancer | Thermo Fisher Scientific | Cat# 21050 |
| [Recombinant Human IL-2](javascript:TrackClick('/zh/recombinant-human-il-2',%20%7b%20list:%20%20'Search%20Results',name:%20'Recombinant%20Human%20IL-2',id:%20'200-02',position:%2010%7d);) | PeproTech | Cat# 200-02 |
| [InVivoMAb anti-mouse CXCL9 (MIG)](http://www.neobioscience.com/prod_view.aspx?TypeId=361&Id=429274&FId=t3:361:3) | Bioxcell | Cat# BE0309 |
| DAPI | Beyotime | Cat# C1006 |
| 4% paraformaldehyde | Beyotime | Cat# P0099 |
| [Triton™ X-100](https://www.sigmaaldrich.cn/CN/zh/substance/tritonx100123459036195) | Sigma-Aldrich | Cat# X100 |
| PMA | Sigma-Aldrich | Ca t#P8139 |
| Opti-MEM | Invitrogen | Cat #31985070 |
| Citrate buffer pH 6.0 | Thermo Fisher Scientific | Cat# 005000 |
| Critical commercial assays |  |  |
| Mouse CXCL9 ELISA kit | R&D Systems | Cat# DY492 |
| Human CXCL9 ELISA kit | R&D Systems | Cat# DY392 |
| Mouse CXCL10 ELISA kit | R&D Systems | Cat# DY466 |
| Human CXCL10 ELISA kit | R&D Systems | Cat# DY266 |
| Pierce BCA Protein Assay | Thermo Fisher Scientific | Cat# 23225 |
| RecoverAll™ Total Nucleic Acid Isolation Kit | Thermo Fisher Scientific | Cat#AM1975 |
| ChIP Assay Kit | Millipore | Cat# 17-295 |
| Hyperactive Universal CUT&Tag Assay Kit for Illumina Pro | Vazyme | Cat#TD904 |
| QuantiNova SYBR PCR Mix Kit | QIAGEN | Cat# 208252 |
| PrimeScript RT Master Mix | TAKARA | Cat# RR036A |
| Zombie Aqua™ Fixable Viability Kit | Biolegend | Cat# 423102 |
| Human Pan T Cell Isolation Kit | Miltenyi Biotec | Cat# 130-096-535 |
| Wizard® Plus SV Minipreps DNA Purification Systems | Promega | Cat# A1460 |
| Wizard® SV Gel and PCR Clean-Up System | Promega | Cat# A9282 |
| PrimeSTAR® Max DNA Polymerase | TAKARA | Cat# R054A |
| Protease/Phosphatase Inhibitor Cocktail | Cell Signaling Technology | Cat# 5872 |
| Dual Luciferase Reporter Assay | Promega | Cat# E1910 |
| Experimental models: Cell lines |  |  |
| BMDMs | This paper | N/A |
| THP-1 | ATCC | Cat# HIB-202 |
| **Deposited data** |  |  |
| LC-MS analysis of metabolites and flux | This paper | MetaboLights: MTBLS3933 ? |
| Experimental models: Organisms/strains |  |  |
| C57BL/6 J mice | Vital River Laboratory Animal  Technology Co. | N/A |
| *Ahr*-^flox/flox^ mice | gifted by Institute of Basic Medical Sciences, Chinese Academy of Medical Sciences |  |
| *Lyz2*-Cre mice | gifted by Institute of Basic Medical Sciences, Chinese Academy of Medical Sciences |  |
| **Software and algorithms** |  |  |
| Flow Jo_v10 | Flowjo | https://www.flowjo.com |
| ImageJ | ImageJ | https://imagej.net/software/ImageJ |
| GraphPad Prism 8.0 | GraphPad Software | https://www.graphpad.com/  scientificsoftware/prism/ |
| SlideViewer | SlideViewer | https://www.3dhistech.com/research/software-downloads/ |

**Table S2. siRNA sequences and primers for qPCR or CHIP-PCR**

| **siRNAs** | **Species** | **Target sequence** |
| --- | --- | --- |
| *NF-κB*-siRNA-1 | human | AAAUAUGGAUCAUCUUCUGCC |
| NF-κB-siRNA-2 | human | AAAUGAAACAUUUGUUCAGGC |
| *CREBBP*-siRNA-1 | human | UGAGUUUGGCUCUUUUGGGGU |
| *CREBBP*-siRNA-2 | human | AAAAUCUGUGCUGUCAUUCGC |
| *AhR*-siRNA-1 | human | GCACCGATGGGAAATGATA |
| *AhR*-siRNA-2 | human | CTTCCAAGCGGCATAGAGA |
| *IDO1*-siRNA-1 | human | GGATGTTCATTGCTAAACA |
| *IDO1*-siRNA-2 | human | GTCTCTCTATTGGTGGAAA |
| *STAT1*-siRNA-1 | human | CTGGATATATCAAGACTGA |
| *STAT1*-siRNA-2 | human | GCACGCTGCCAATGATGTT |
| *SOCS3*-siRNA-1 | human | CAGCATCTCTGTCGGAAGA |
| *SOCS3*-siRNA-2 | human | GAAGAGCCTATTACATCTA |
| *SOCS3*-siRNA-1 | mouse | TCTTCACGTTGAGCGTCAA |
| *SOCS3*-siRNA-2 | mouse | GCATCTTTGTCGGAAGACT |
| *IDO1*-siRNA-1 | mouse | GCCTCCTATTCTGTCTTAT |
| *IDO1*-siRNA-2 | mouse | GCACTGCACGACATAGCTA |
|  |  |  |
| **Real-Time PCR primer** | **Species** | **Sequence 5’-3’** |
| *IDO1*-Forward | human | GCCAGCTTCGAGAAAGAGTTG |
| *IDO1*-Reverse | human | ATCCCAGAACTAGACGTGCAA |
| *IDO1*-Forward | mouse | TGGCGTATGTGTGGAACCG |
| *IDO1*-Reverse | mouse | CTCGCAGTAGGGAACAGCAA |
| *IDO2*-Forward | human | CCACAGACCGAATGTGAAGAC |
| *IDO2*-Reverse | human | TGTTGGCAATTTCCATCCAAGG |
| *IDO2*-Forward | mouse | TCAAAGTCAGAGCATGACGCT |
| *IDO2*-Reverse | mouse | GGCGGTTCTCGATTAAGTGAG |
| *TDO2*-Forward | human | AAGGTTGTTTCTCGGATGCAC |
| *TDO2*-Reverse | human | TGTCATCGTCTCCAGAATGGAA |
| *TDO2*-Forward | mouse | TGGCAATTACTTGCAGTTGGA |
| *TDO2*-Reverse | mouse | GTGCTCGTCATGGATTTTGTTC |
| *CXCL9*-Forward | human | CCAGTAGTGAGAAAGGGTCGC |
| *CXCL9*-Reverse | human | AGGGCTTGGGGCAAATTGTT |
| *CXCL9*-Forward | mouse | GGAGTTCGAGGAACCCTAGTG |
| *CXCL9*-Reverse | mouse | GGGATTTGTAGTGGATCGTGC |
| *CXCL10*-Forward | human | GTGGCATTCAAGGAGTACCTC |
| *CXCL10*-Reverse | human | TGATGGCCTTCGATTCTGGATT |
| *CXCL10*-Forward | mouse | CCAAGTGCTGCCGTCATTTTC |
| *CXCL10*-Reverse | mouse | GGCTCGCAGGGATGATTTCAA |
| *CCL1*-Forward | human | CTCATTTGCGGAGCAAGAGAT |
| *CCL1*-Reverse | human | GCCTCTGAACCCATCCAACTG |
| *CCL2*-Forward | human | CAGCCAGATGCAATCAATGCC |
| *CCL2*-Reverse | human | TGGAATCCTGAACCCACTTCT |
| *CCL3*-Forward | human | AGTTCTCTGCATCACTTGCTG |
| *CCL3*-Reverse | human | CGGCTTCGCTTGGTTAGGAA |
| *CCL4*-Forward | human | CTGTGCTGATCCCAGTGAATC |
| *CCL4*-Reverse | human | TCAGTTCAGTTCCAGGTCATACA |
| *CCL5*-Forward | human | CCAGCAGTCGTCTTTGTCAC |
| *CCL5*-Reverse | human | CTCTGGGTTGGCACACACTT |
| *CYP1A1*-Forward | human | TCGGCCACGGAGTTTCTTC |
| *CYP1A1*-Reverse | human | GGTCAGCATGTGCCCAATCA |
| *CYP1A1*-Forward | mouse | CAATGAGTTTGGGGAGGTTACTG |
| *CYP1A1*-Reverse | mouse | CCCTTCTCAAATGTCCTGTAGTG |
| *CYP1B1*-Forward | human | TGAGTGCCGTGTGTTTCGG |
| *CYP1B1*-Reverse | human | GTTGCTGAAGTTGCGGTTGAG |
| *CYP1B1*-Forward | mouse | CCACCAGCCTTAGTGCAGAC |
| *CYP1B1*-Reverse | mouse | GGCCAGGACGGAGAAGAGT |
| *AHR*-Forward | human | ACATCACCTACGCCAGTCG |
| *AHR*-Reverse | human | CGCTTGGAAGGATTTGACTTGA |
| *AHR*-Forward | mouse | GCCCTTCCCGCAAGATGTTAT |
| *AHR*-Reverse | mouse | TCAGCAGGGGTGGACTTTAAT |
| *SOCS1*-Forward | human | CACGCACTTCCGCACATTC |
| *SOCS1*-Reverse | human | TAAGGGCGAAAAAGCAGTTCC |
| *SOCS2*-Forward | human | TTAAAAGAGGCACCAGAAGGAAC |
| *SOCS2*-Reverse | human | AGTCGATCAGATGAACCACACT |
| *SOCS3*-Forward | human | CCTGCGCCTCAAGACCTTC |
| *SOCS3*-Reverse | human | GTCACTGCGCTCCAGTAGAA |
| *SOCS3*-Forward | mouse | TGCGCCTCAAGACCTTCAG |
| *SOCS3*-Reverse | mouse | GCTCCAGTAGAATCCGCTCTC |
| *SOCS4*-Forward | human | GCCGACAGAAAAGACGGTTAT |
| *SOCS4*-Reverse | human | GGTTCCTTAAAGACACTTCGGTT |
| *SOCS5*-Forward | human | GTGCCACAGAAATCCCTCAAA |
| *SOCS5*-Reverse | human | TCTCTTCGTGCAAGTCTTGTTC |
| *SOCS6*-Forward | human | TGTGCCTGTCGTTATTGGACT |
| *SOCS6*-Reverse | human | CCAAAGGATACATCCCCTCATCT |
| *SOCS7*-Forward | human | GGGTCAAGACAGTCGGTGG |
| *SOCS7*-Reverse | human | TCTCGGCCTCCGATTCCAA |
| *CISH*-Forward | human | GAACTGCCCAAGCCAGTCAT |
| *CISH*-Reverse | human | GCTATGCACAGCAGATCCTCC |
| *GAPDH*-Forward | human | GGAGCGAGATCCCTCCAAAAT |
| *GAPDH*-Reverse | human | GGCTGTTGTCATACTTCTCATGG |
| *GAPDH*-Forward | mouse | AGGTCGGTGTGAACGGATTTG |
| *GAPDH*-Reverse | mouse | GGGGTCGTTGATGGCAACA |
|  |  |  |
| **CHIP-PCR primer** | **Species** | **Sequence 5’-3’** |
| *SOCS3-ChIP*-Forward | human | TCCGGAAATTCTCTCCTGCT |
| *SOCS3-ChIP*-Reverse | human | CTAGGGGAGGGCAGTTTGG |

**Table S3. Clinical information of healthy people for Figure1A**

| NO. | gender | age | Sample |
| --- | --- | --- | --- |
| 1 | M | 40 | Peripheral blood |
| 2 | M | 36 | Peripheral blood |
| 3 | F | 32 | Peripheral blood |
| 4 | F | 34 | Peripheral blood |
| 5 | M | 32 | Peripheral blood |
| 6 | F | 27 | Peripheral blood |
| 7 | M | 26 | Peripheral blood |
| 8 | F | 30 | Peripheral blood |
| 9 | F | 25 | Peripheral blood |
| 10 | F | 24 | Peripheral blood |

**Table S4. Clinical information of people with TB for Figure1A**

| Patients NO. | gender | age | Status | Sample | diagnose |
| --- | --- | --- | --- | --- | --- |
| 1 | M | 37 | Newly diagnosis | Peripheral blood | secondary pulmonary tuberculosis |
| 2 | M | 57 | Newly diagnosis | Peripheral blood | secondary pulmonary tuberculosis |
| 3 | F | 76 | Newly diagnosis | Peripheral blood | secondary pulmonary tuberculosis |
| 4 | M | 33 | Newly diagnosis | Peripheral blood | secondary pulmonary tuberculosis |
| 5 | F | 68 | Newly diagnosis | Peripheral blood | secondary pulmonary tuberculosis |
| 6 | F | 70 | Newly diagnosis | Peripheral blood | secondary pulmonary tuberculosis |
| 7 | F | 32 | Newly diagnosis | Peripheral blood | secondary pulmonary tuberculosis |
| 8 | M | 72 | Newly diagnosis | Peripheral blood | secondary pulmonary tuberculosis |
| 9 | F | 61 | Newly diagnosis | Peripheral blood | secondary pulmonary tuberculosis |
| 10 | F | 33 | Newly diagnosis | Peripheral blood | secondary pulmonary tuberculosis |
| 11 | F | 25 | Newly diagnosis | Peripheral blood | secondary pulmonary tuberculosis |
| 12 | M | 44 | Newly diagnosis | Peripheral blood | secondary pulmonary tuberculosis |
| 13 | F | 26 | Newly diagnosis | Peripheral blood | secondary pulmonary tuberculosis |
| 14 | F | 49 | Newly diagnosis | Peripheral blood | secondary pulmonary tuberculosis |
| 15 | M | 10 | Newly diagnosis | Peripheral blood | secondary pulmonary tuberculosis |
| 16 | M | 60 | Newly diagnosis | Peripheral blood | secondary pulmonary tuberculosis |
| 17 | F | 72 | Newly diagnosis | Peripheral blood | secondary pulmonary tuberculosis |
| 18 | F | 30 | Newly diagnosis | Peripheral blood | secondary pulmonary tuberculosis |
| 19 | F | 32 | Newly diagnosis | Peripheral blood | secondary pulmonary tuberculosis |
| 20 | F | 30 | Newly diagnosis | Peripheral blood | secondary pulmonary tuberculosis |
| 21 | F | 55 | Newly diagnosis | Peripheral blood | secondary pulmonary tuberculosis |
| 22 | M | 58 | Newly diagnosis | Peripheral blood | secondary pulmonary tuberculosis |
| 23 | M | 68 | Newly diagnosis | Peripheral blood | secondary pulmonary tuberculosis |
| 24 | F | 52 | Newly diagnosis | Peripheral blood | secondary pulmonary tuberculosis |
| 25 | F | 60 | Newly diagnosis | Peripheral blood | secondary pulmonary tuberculosis |
| 26 | M | 27 | Newly diagnosis | Peripheral blood | secondary pulmonary tuberculosis |

**Table S5. Clinical information of healthy people for Figure1B-D**

| Patients NO. | gender | age | Status | Sample | diagnose |
| --- | --- | --- | --- | --- | --- |
| 1 | F | 25 | Newly diagnosis | Paraffin section and Peripheral blood | Secondary pulmonary tuberculosis |
| 2 | M | 44 | Newly diagnosis | Paraffin section and Peripheral blood | secondary pulmonary tuberculosis |
| 3 | F | 26 | Newly diagnosis | Paraffin section and Peripheral blood | secondary pulmonary tuberculosis |
| 4 | F | 49 | Newly diagnosis | Paraffin section and Peripheral blood | secondary pulmonary tuberculosis |
| 5 | M | 10 | Newly diagnosis | Paraffin section and Peripheral blood | secondary pulmonary tuberculosis |
| 6 | M | 60 | Newly diagnosis | Paraffin section and Peripheral blood | secondary pulmonary tuberculosis |
| 7 | F | 72 | Newly diagnosis | Paraffin section and Peripheral blood | secondary pulmonary tuberculosis |
| 8 | F | 30 | Newly diagnosis | Paraffin section and Peripheral blood | secondary pulmonary tuberculosis |
| 9 | F | 32 | Newly diagnosis | Paraffin section and Peripheral blood | secondary pulmonary tuberculosis |
| 10 | F | 30 | Newly diagnosis | Paraffin section and Peripheral blood | secondary pulmonary tuberculosis |
| 11 | F | 55 | Newly diagnosis | Paraffin section and Peripheral blood | secondary pulmonary tuberculosis |
| 12 | M | 58 | Newly diagnosis | Paraffin section and Peripheral blood | secondary pulmonary tuberculosis |
| 13 | M | 68 | Newly diagnosis | Paraffin section and Peripheral blood | secondary pulmonary tuberculosis |
| 14 | F | 52 | Newly diagnosis | Paraffin section and Peripheral blood | secondary pulmonary tuberculosis |
| 15 | F | 60 | Newly diagnosis | Paraffin section and Peripheral blood | secondary pulmonary tuberculosis |
| 16 | M | 27 | Newly diagnosis | Paraffin section and Peripheral blood | secondary pulmonary tuberculosis |

**Table S6. Clinical information of people with TB for Figure1E and Figure1F**

| Patients NO. | gender | age | Status | Sample | diagnose |
| --- | --- | --- | --- | --- | --- |
| 1 | F | 68 | Newly diagnosis | Peripheral blood | secondary pulmonary tuberculosis |
| 2 | M | 58 | Newly diagnosis | Peripheral blood | secondary pulmonary tuberculosis |
| 3 | M | 37 | Newly diagnosis | Peripheral blood | secondary pulmonary tuberculosis |
| 4 | F | 41 | Newly diagnosis | Peripheral blood | secondary pulmonary tuberculosis |
| 5 | M | 70 | Newly diagnosis | Peripheral blood | secondary pulmonary tuberculosis |
| 6 | F | 54 | Newly diagnosis | Peripheral blood | secondary pulmonary tuberculosis |
| 7 | M | 37 | Newly diagnosis | Peripheral blood | secondary pulmonary tuberculosis |
| 8 | F | 61 | Newly diagnosis | Peripheral blood | secondary pulmonary tuberculosis |
| 9 | F | 75 | Newly diagnosis | Peripheral blood | secondary pulmonary tuberculosis |
| 10 | M | 70 | Newly diagnosis | Peripheral blood | secondary pulmonary tuberculosis |
| 11 | M | 71 | Newly diagnosis | Peripheral blood | secondary pulmonary tuberculosis |
| 12 | F | 17 | Newly diagnosis | Peripheral blood | secondary pulmonary tuberculosis |
