# Supplementary material for: Kynurenine-AhR reduces T-cell infiltration and induces a delayed T-cell immune response by suppressing the STAT1-CXCL9/CXCL10 axis in tuberculosis
Source: Cell Mol Immunol. 2024 Oct 22;21(12):1426–40. doi: 10.1038/s41423-024-01230-1 (PMC11607402; doi:10.1038/s41423-024-01230-1)

Figure 2C

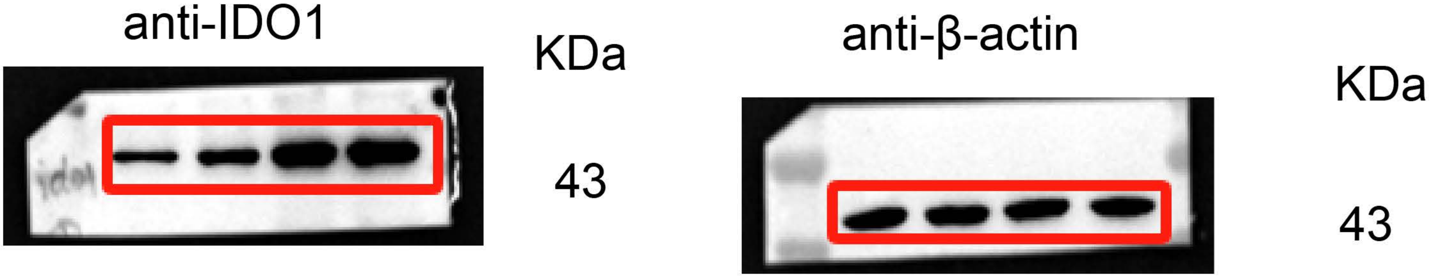

Figure 2D

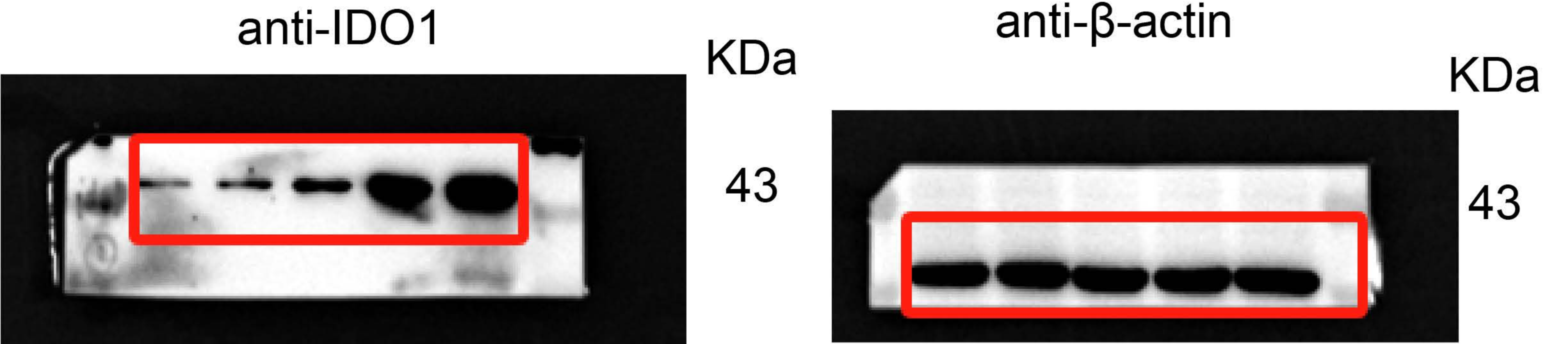

Figure 3D

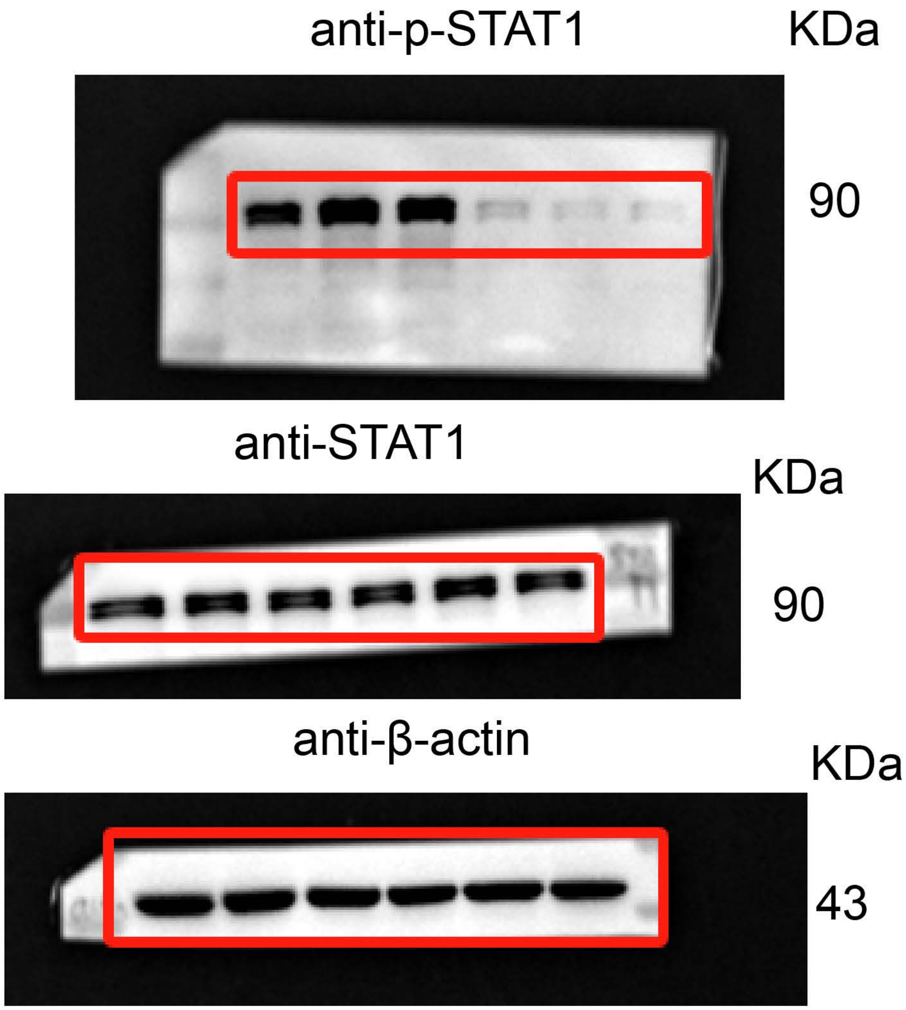

Figure 3E

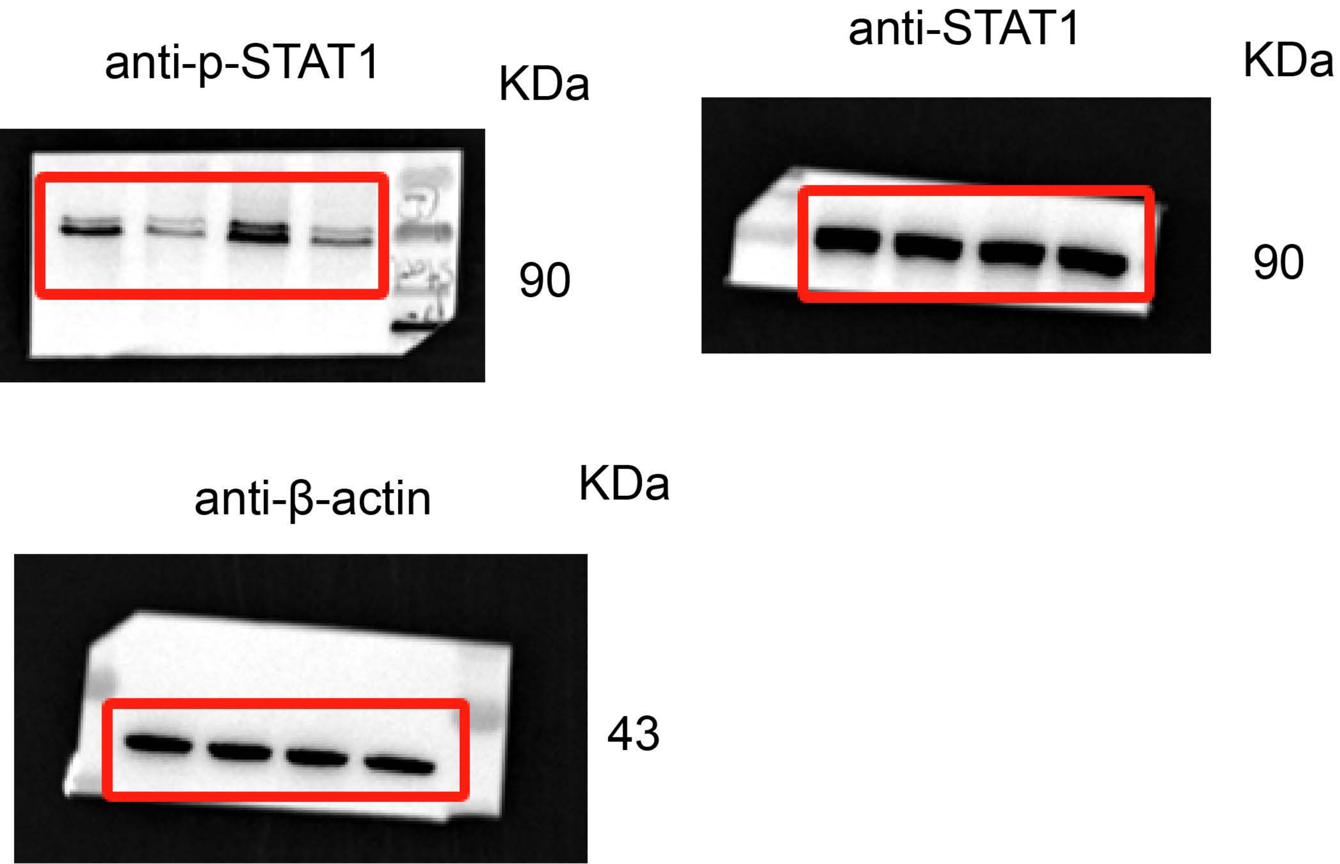

Figure 4B

anti-AhR

KDa

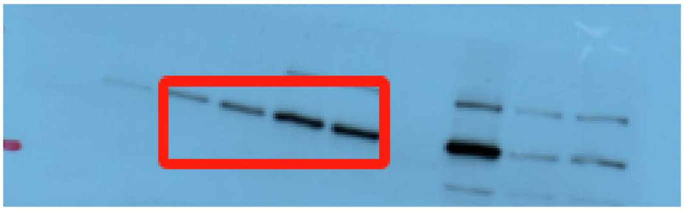

96

anti-α-Tubulin

KDa

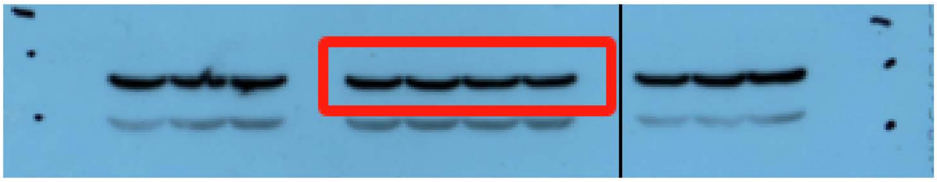

56

Figure 4E

anti-AhR

KDa

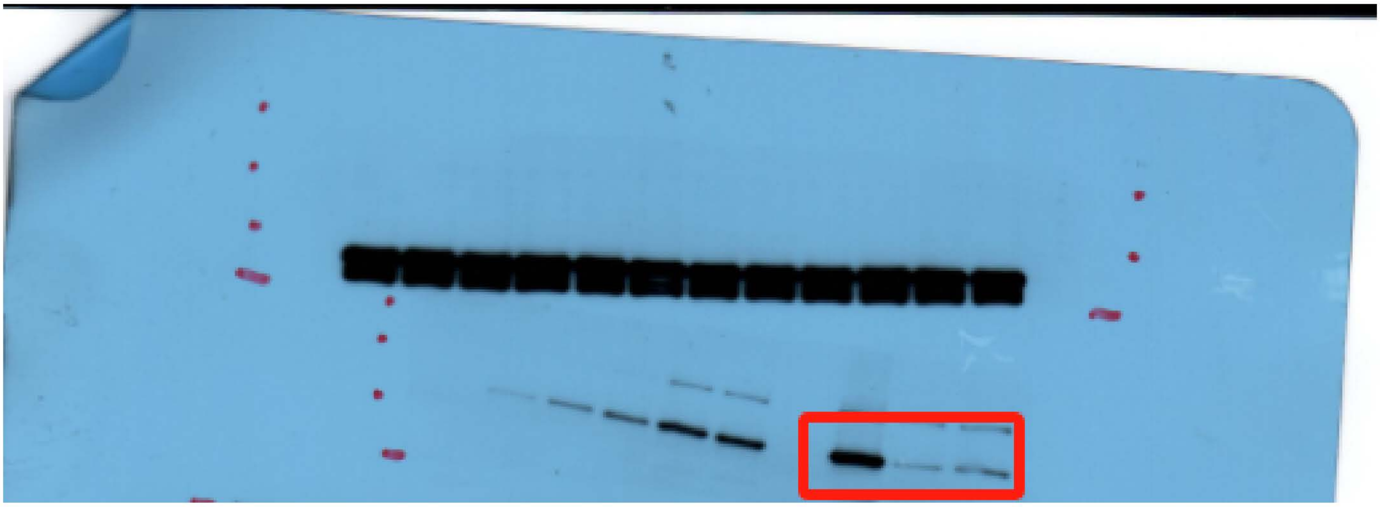

96

anti-α-tublin

KDa

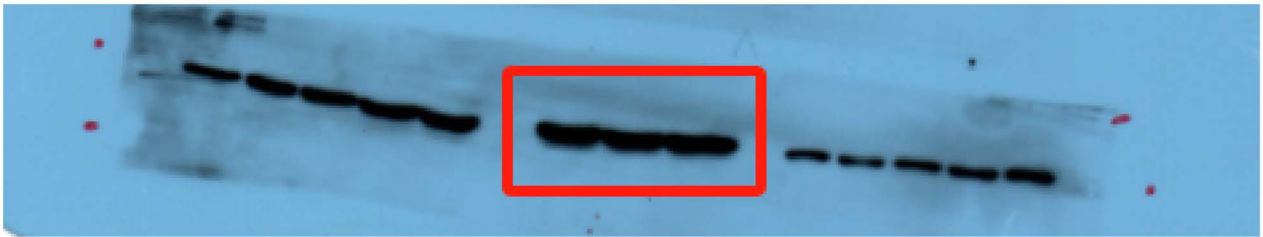

56

Figure 5G

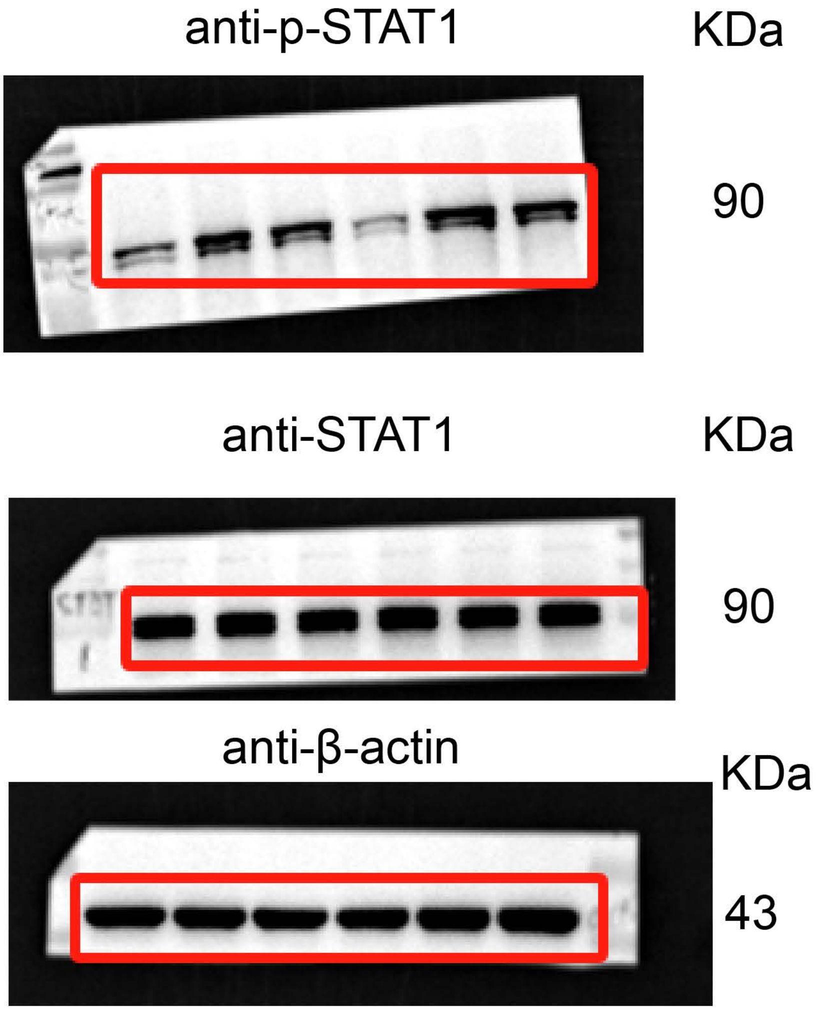

Figure 5H

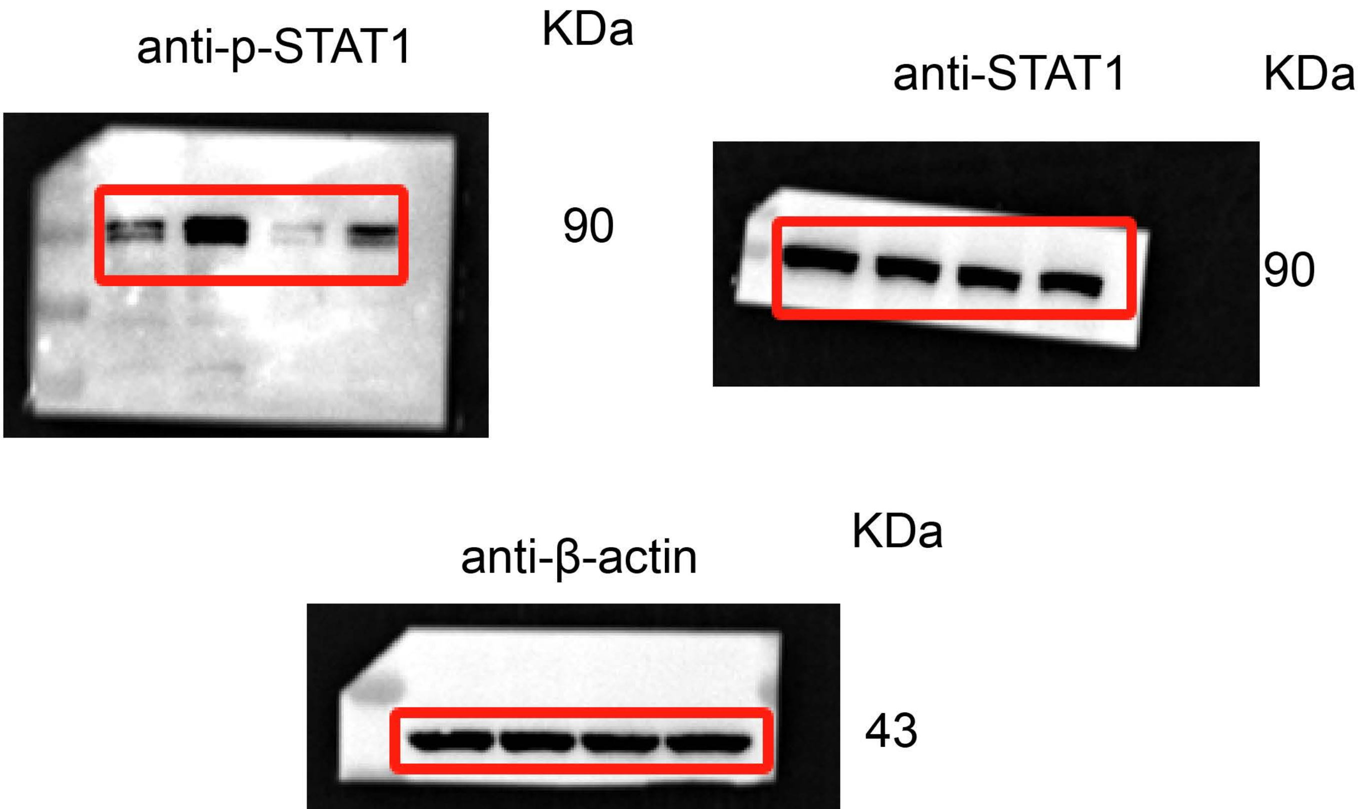

Figure 6E

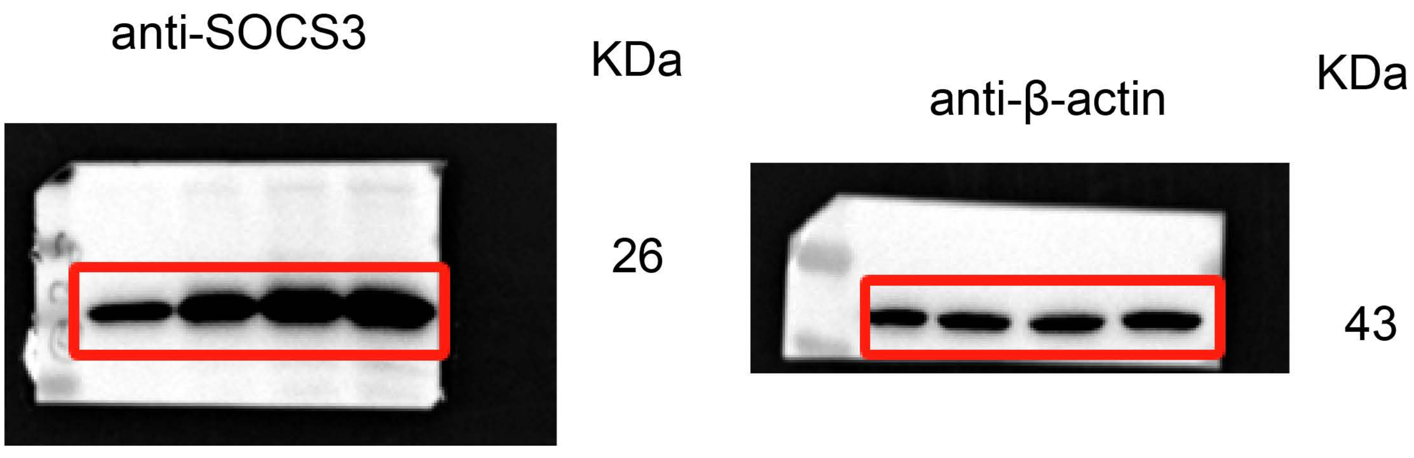

Figure 6F

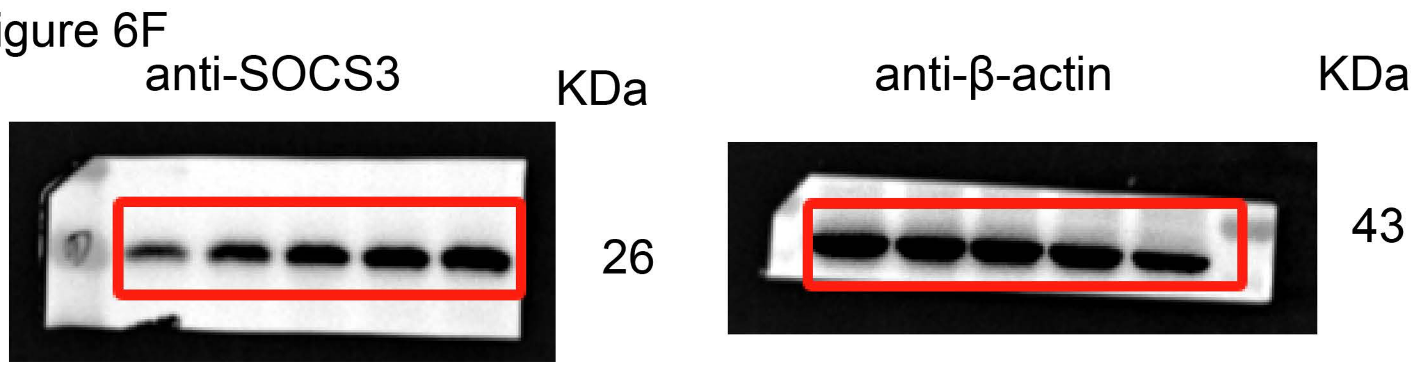

Figure 6G

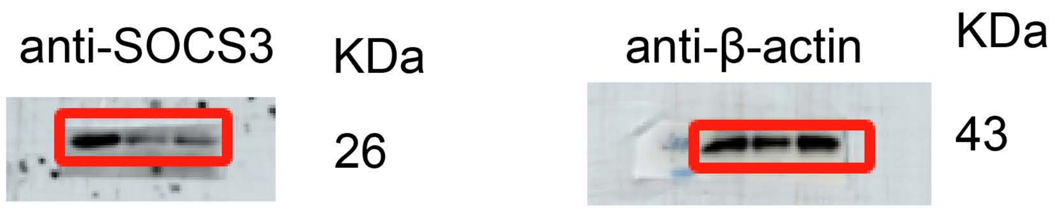

Figure 6J

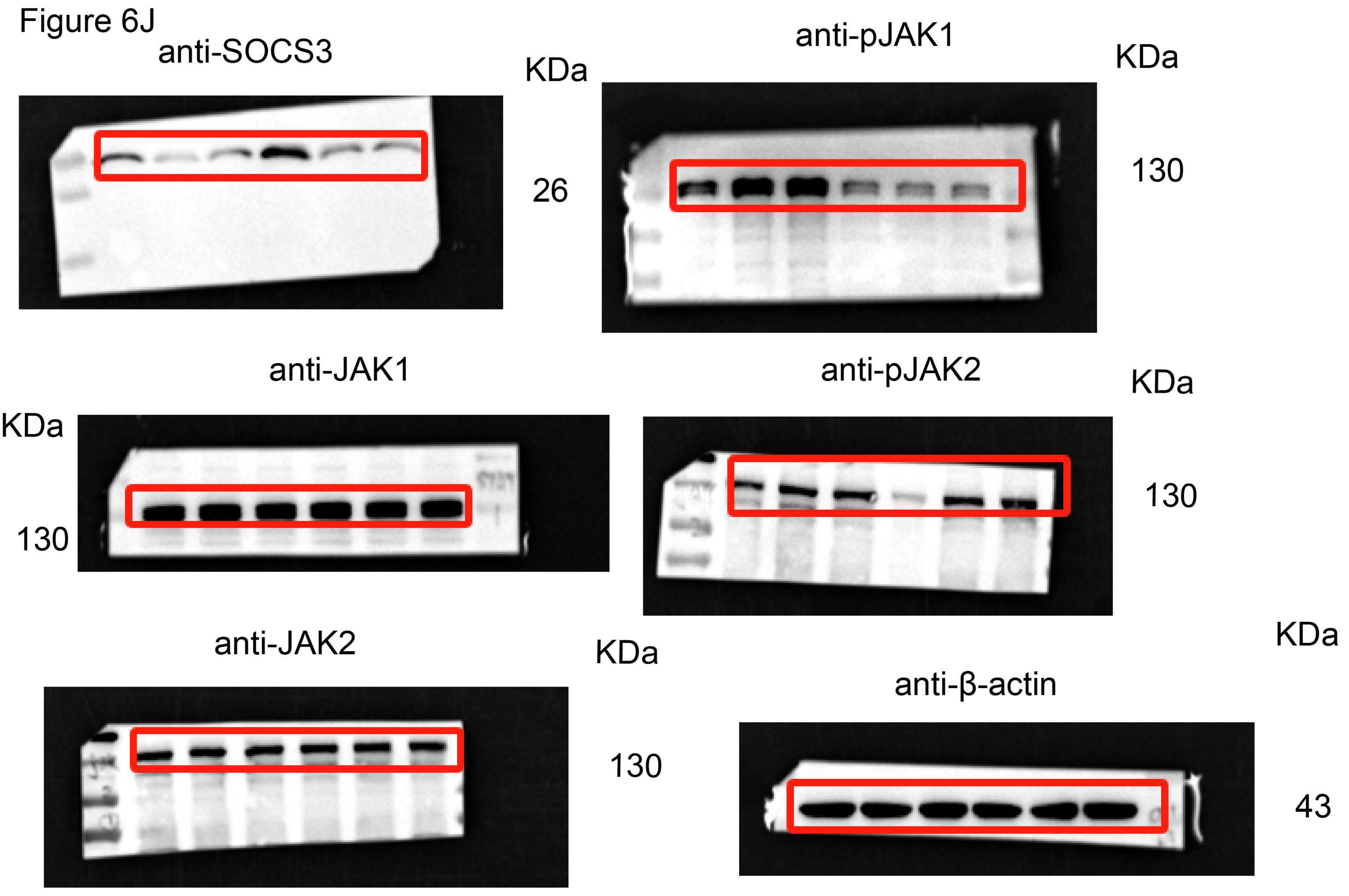

Figure 6K

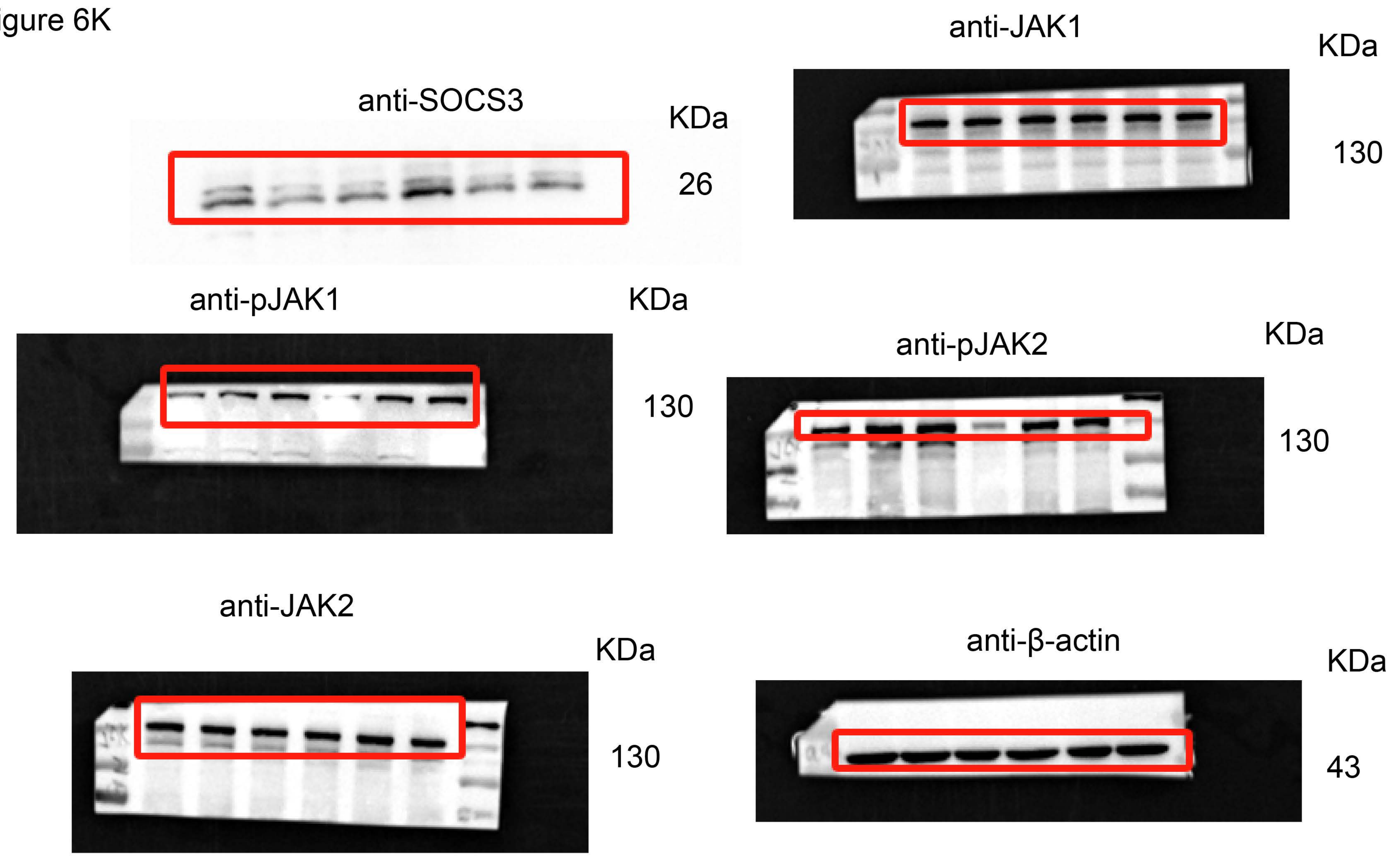

Figure 6L

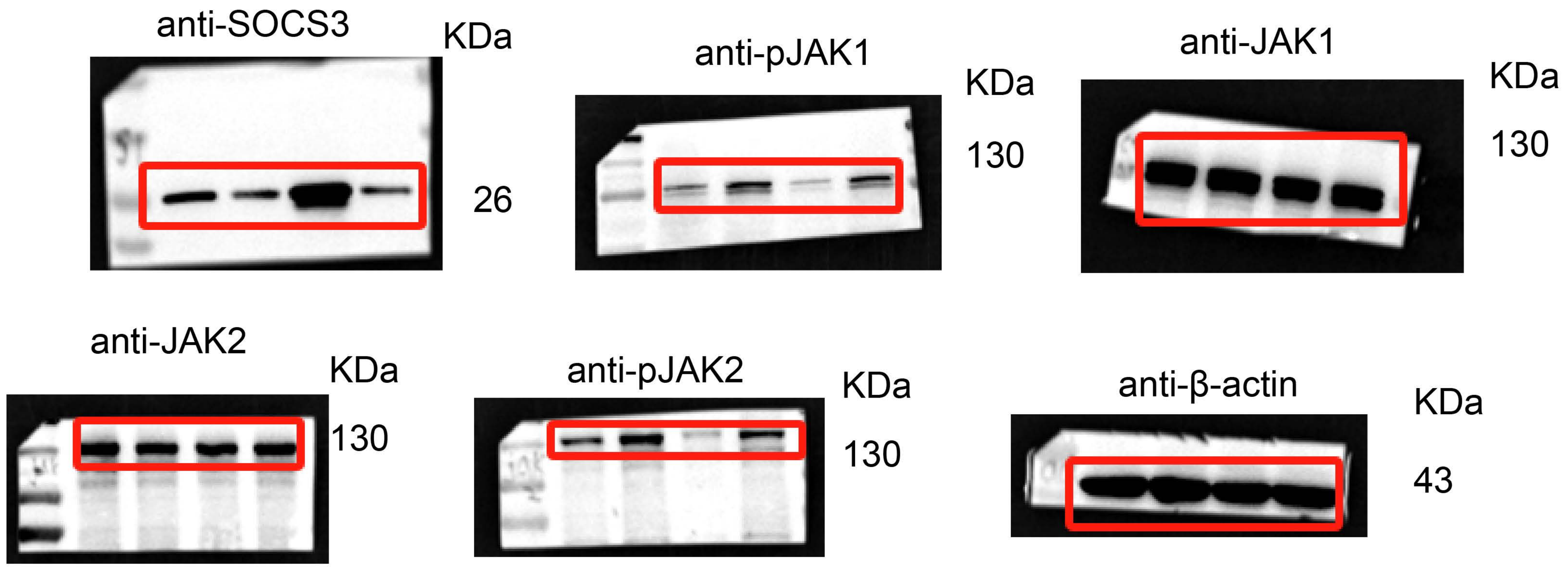

Figure 6M

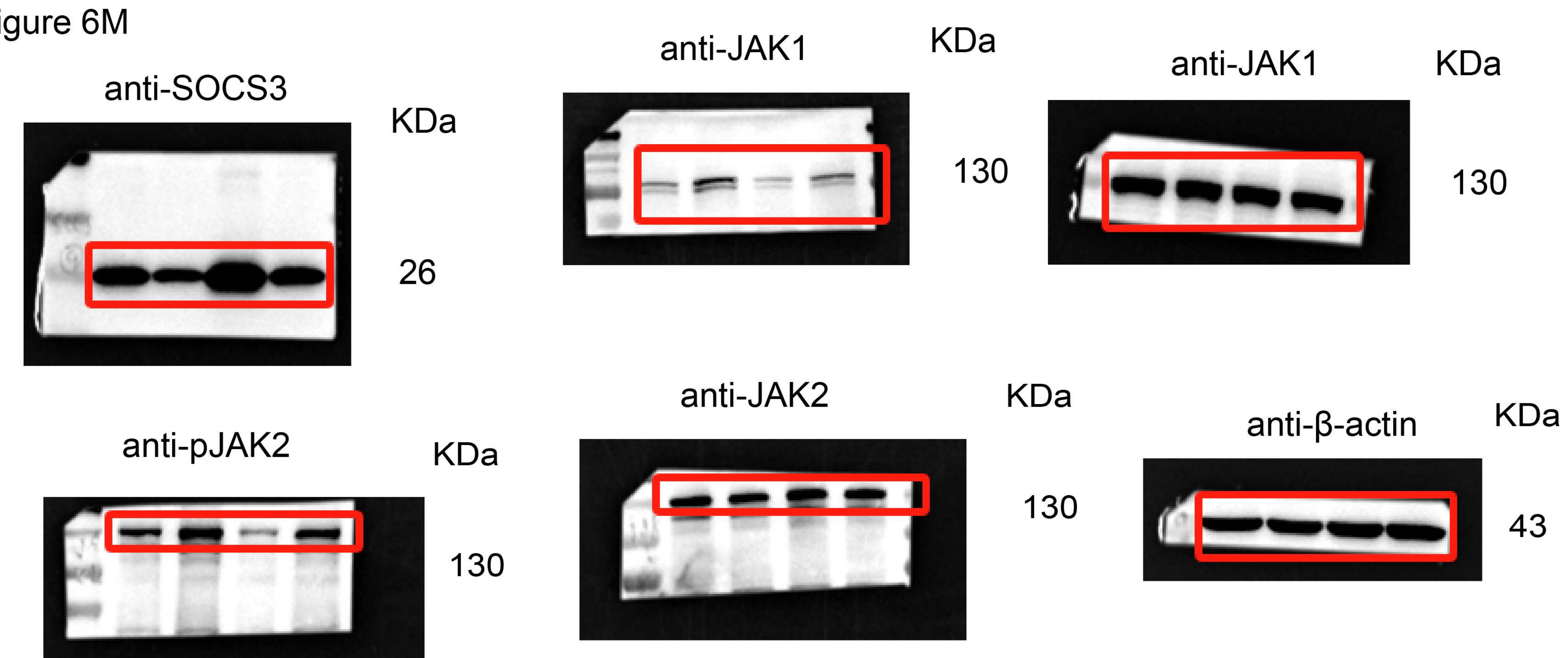

Figure S1B

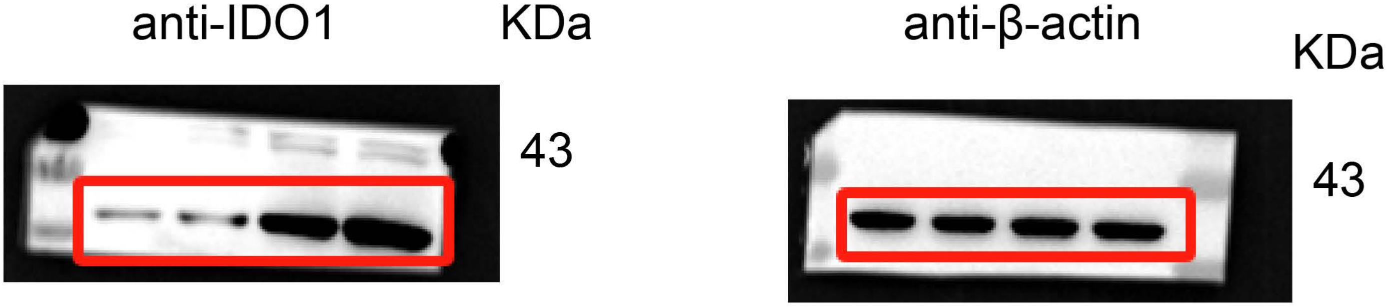

Figure S1C

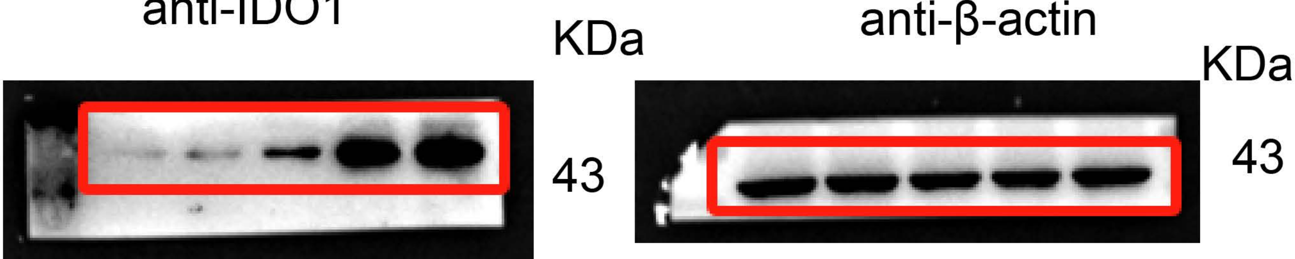

FigureS2C

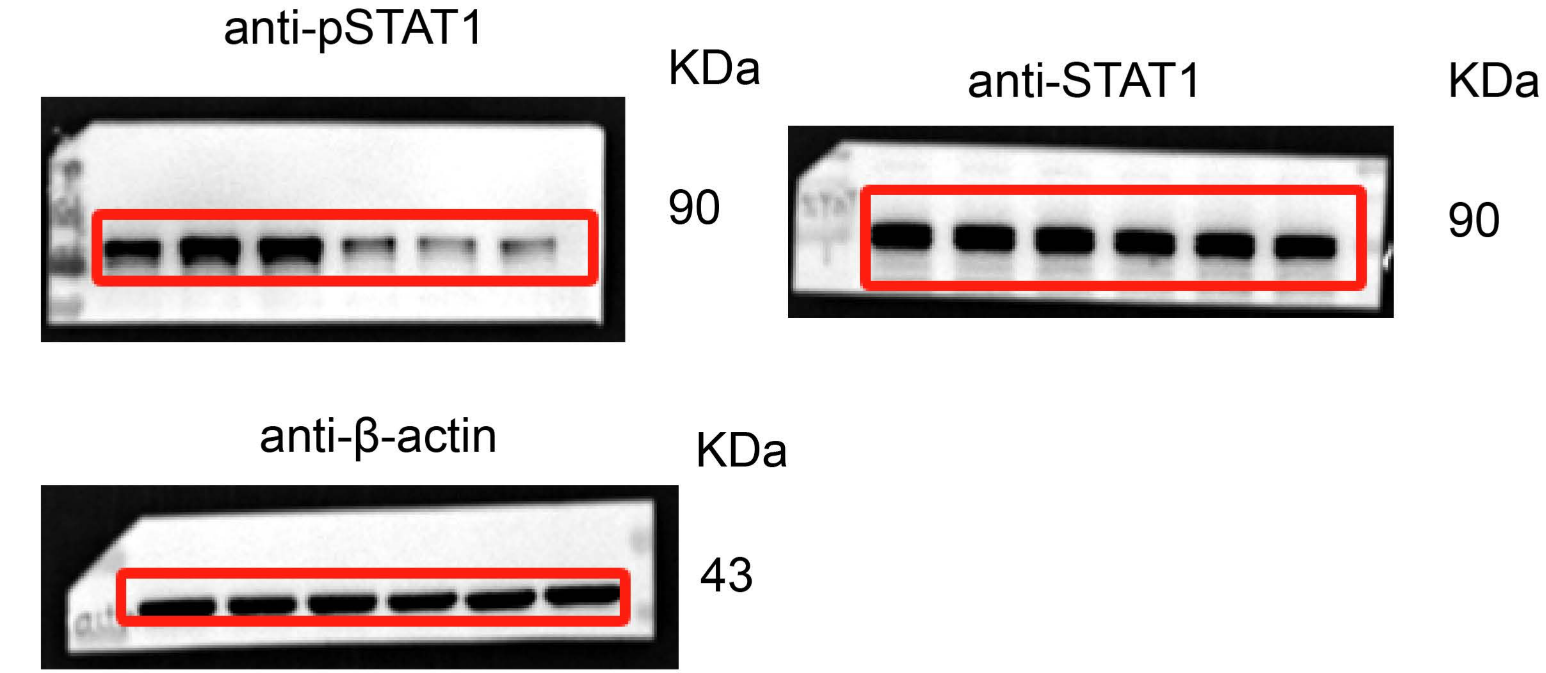

Figure S2D

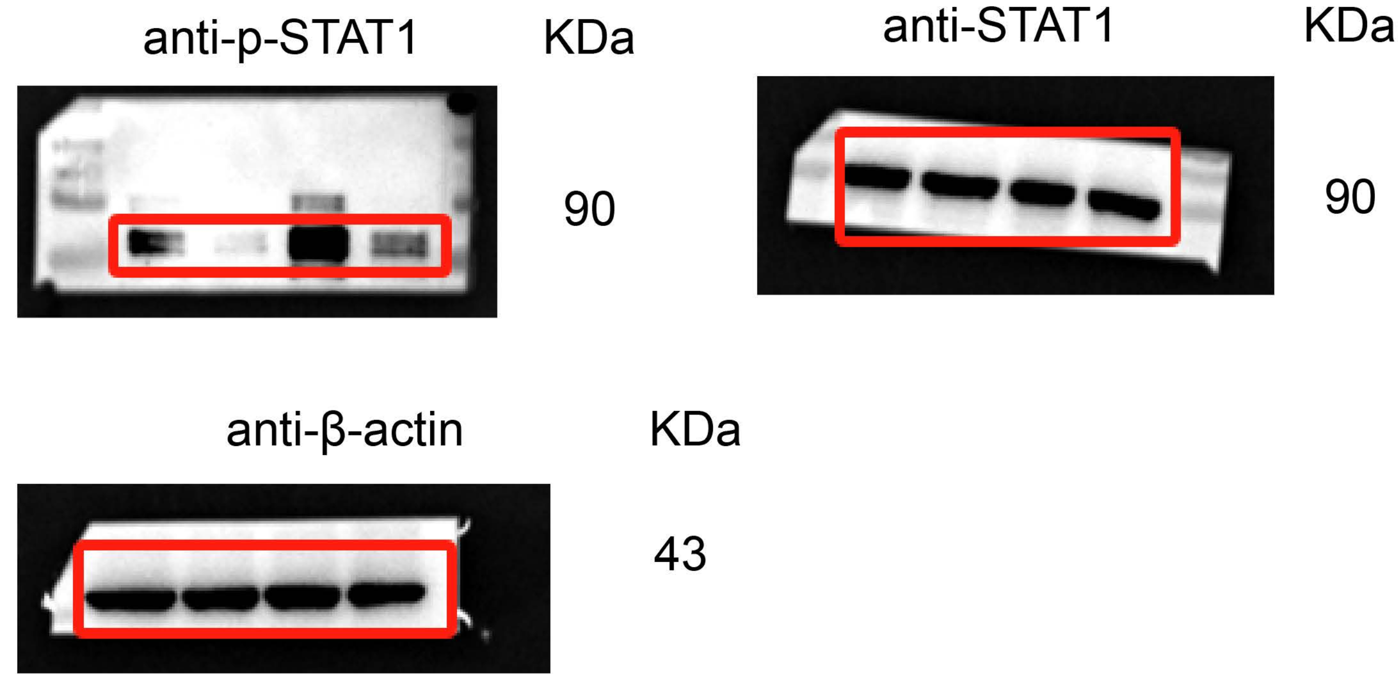

Figure S3I

anti-p-STAT1 KDa

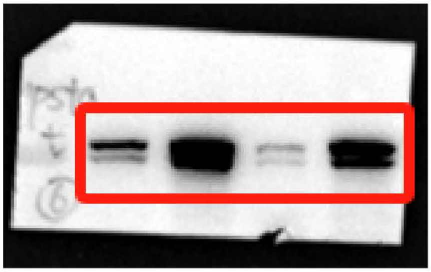

90

anti-STAT1 KDa

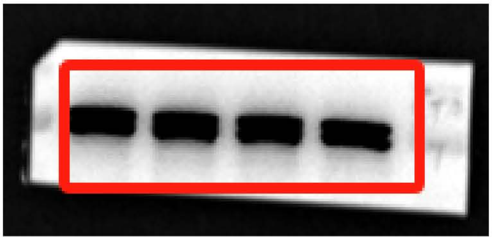

90

anti-β-actin KDa

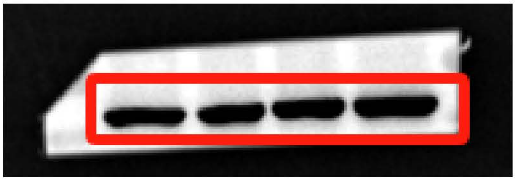

43

Figure3J

anti-p-STAT1 KDa

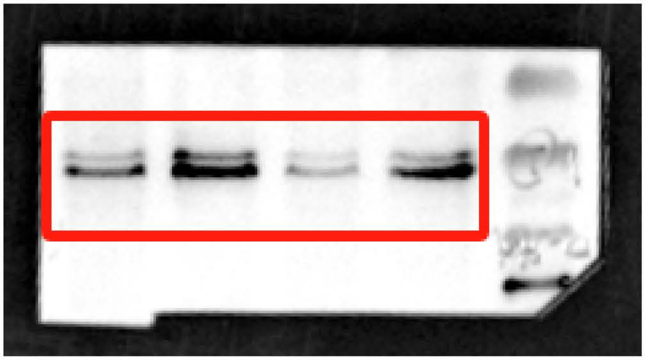

90

anti-STAT1 KDa

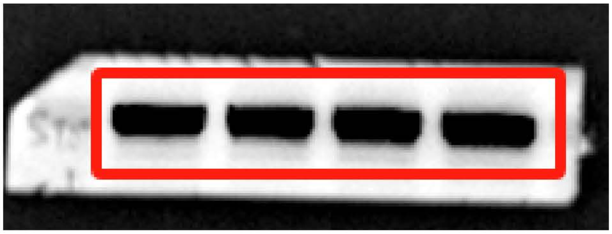

90

anti-β-actin KDa

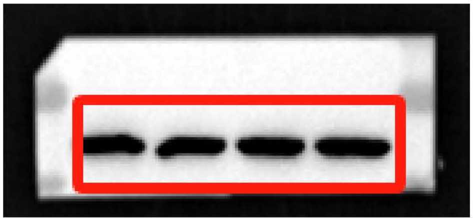

43

Figure S4A

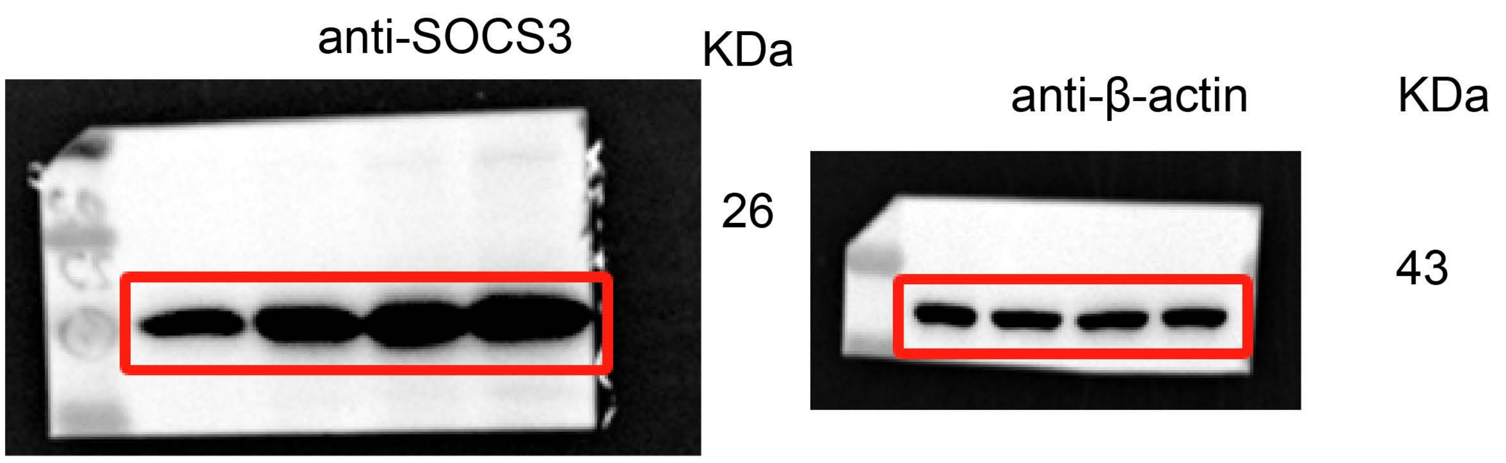

Figure S4B

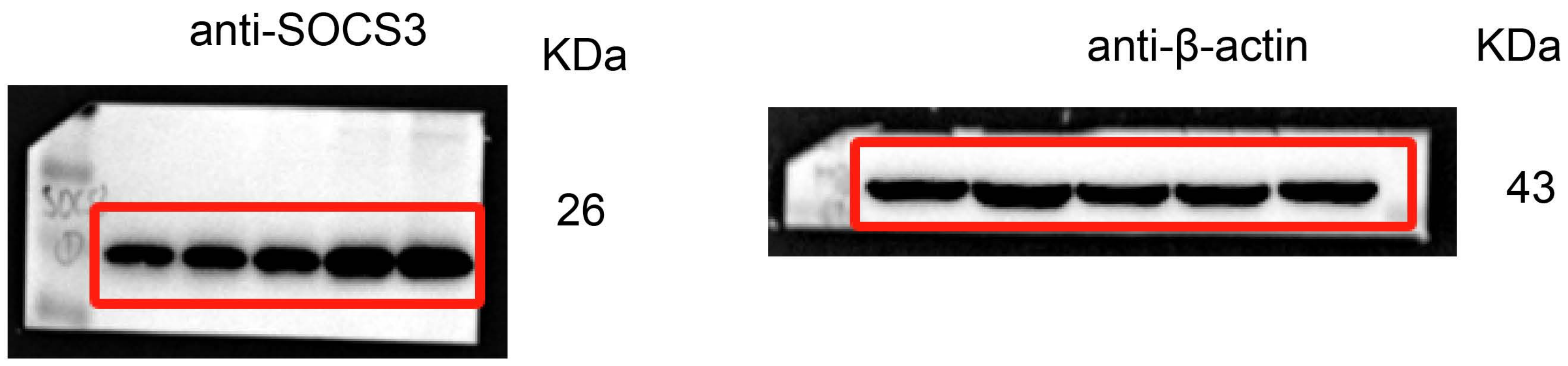

Figure S4E

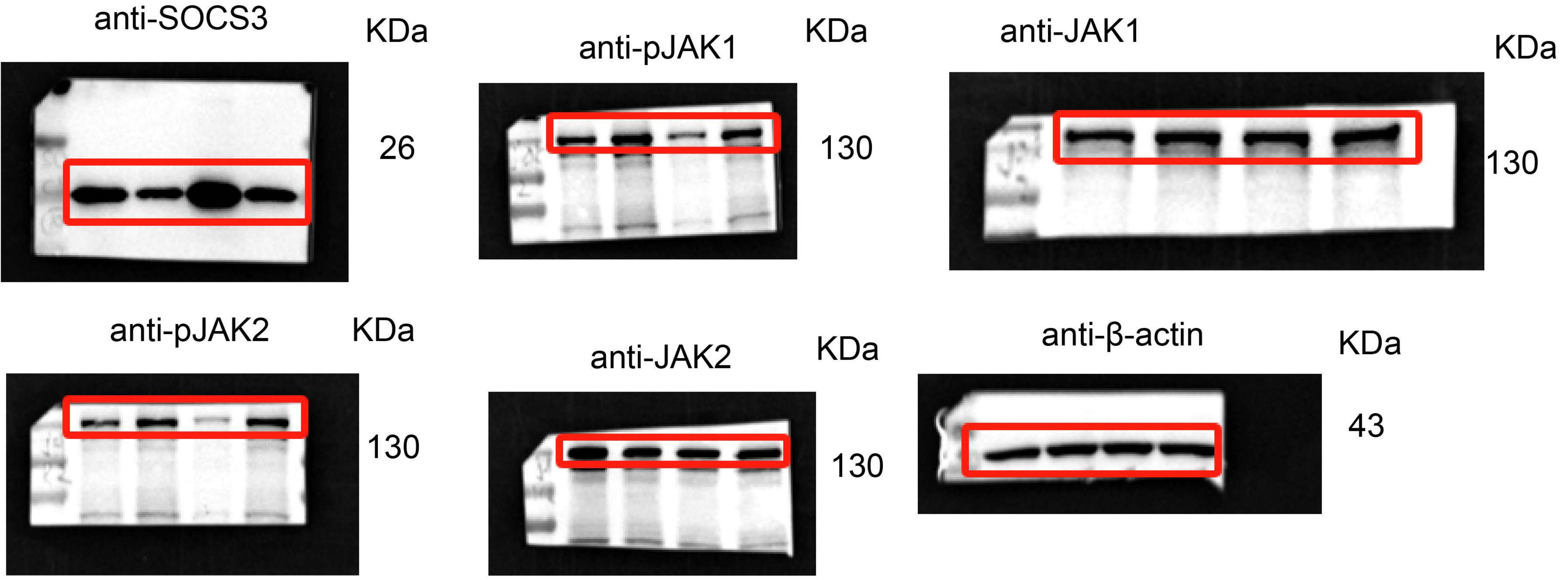

Figure S4F

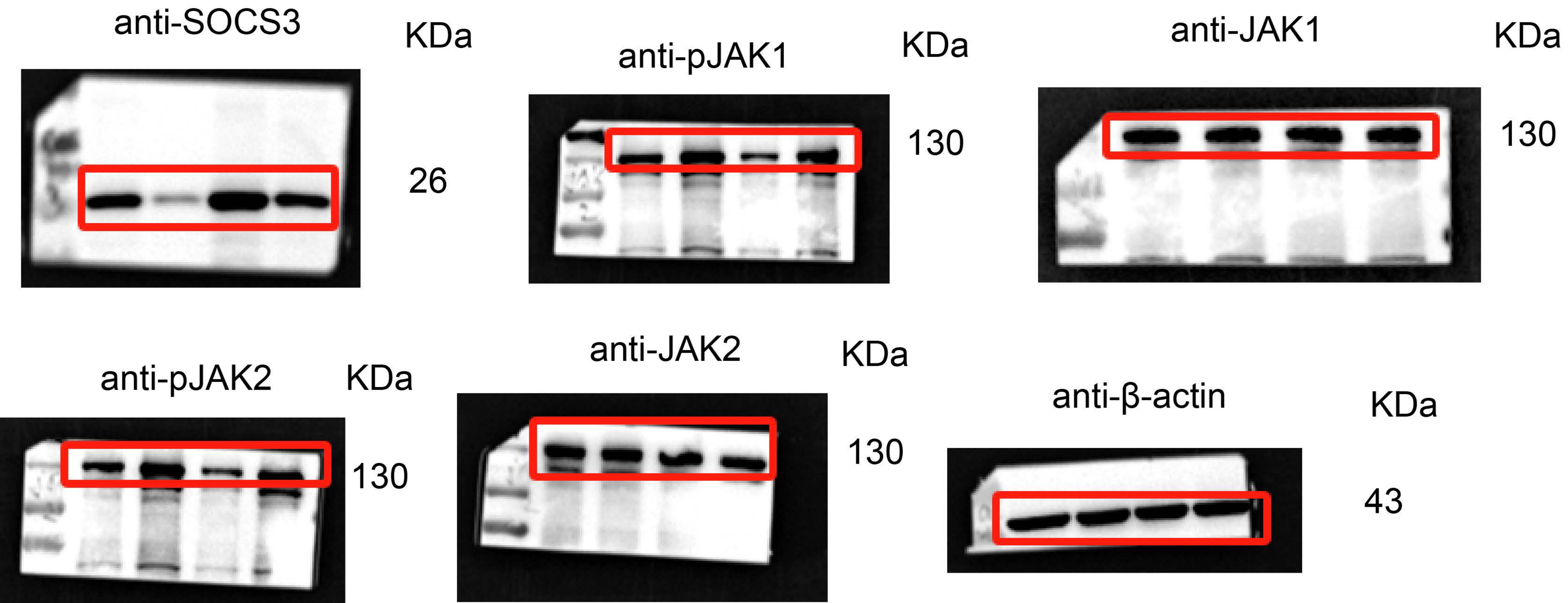

Supplement: Supplementary file 2 — Uncropped images of all western blot [file 41423_2024_1230_MOESM2_ESM.pdf]
